# The cost of drug repurposing: parallel economic evaluation of mirtazapine for severe breathlessness in the multinational BETTER-B trial

**DOI:** 10.1186/s12913-025-13605-9

**Published:** 2025-11-04

**Authors:** Peter May, Charles Normand, Adejoke O. Oluyase, Samantha Smith, Sarah T. Brown, Matthew Maddocks, Massimo Costantini, Sabrina Bajwah, Kathrin Kahnert, Steffen T. Simon, Karen Ryan, David C. Currow, Miriam J. Johnson, Simon P. Hart, Małgorzata Krajnik, Silvia Tanzi, Charlotte E. Bolton, Piotr Janowiak, Elena Turola, Caroline J. Jolley, Hannah Mather, Geraldine Murden, Luca Ghirotto, Bobbie Farsides, Julia M. Brown, Irene J. Higginson

**Affiliations:** 1 Cicely Saunders Institute of Palliative Care, Policy and Rehabilitation, Florence Nightingale Faculty of Nursing, Midwifery & Palliative Care, King’s College London, Bessemer Road, London, SE5 9PJ UK; 2 School of Medicine, Trinity College Dublin, Dublin, Ireland; 3 Clinical Trials Research Unit (CTRU), Leeds Institute of Clinical Trials Research (LICTR), University of Leeds, Leeds, UK; 4 King’s College Hospital NHS Foundation Trust, Bessemer Road, London, UK; 5 Department of Palliative Medicine, LMU University Hospital, LMU Munich, Munich, Germany; 6 MediCenter Germering, Germering, Germany; 7 Department of Palliative Medicine and Center for Integrated Oncology Aachen Bonn Cologne Duesseldorf (CIO ABCD), Faculty of Medicine and University Hospital, University of Cologne, Cologne, Germany; 8 University College Dublin and Mater Misericordiae University Hospital Dublin, Dublin, Ireland; 9 Flinders Ageing Alliance, Flinders University, South Australia, Australia; 10 Wolfson Palliative Care Research Centre, Hull York Medical School, The University of Hull, Hull, UK; 11 Respiratory Research Group, Hull York Medical School, University of Hull, Hull, UK; 12 Department of Palliative Care, Collegium Medicum in Bydgoszcz, Nicolaus Copernicus University in Toruń, Bydgoszcz, Poland; 13 Azienda USL-IRCCS di Reggio Emilia, Reggio Emilia, Italy; 14 NIHR Nottingham Biomedical Research Centre, Translational Medical Sciences, School of Medicine, The University of Nottingham, Nottingham, UK; 15 Division of Pulmonology, Medical University of Gdańsk, Gdańsk, Poland; 16 Centre for Human & Applied Physiological Sciences, School of Basic & Medical Biosciences, King’s College London, London, UK; 17 Brighton and Sussex Medical School, University of Sussex, Brighton, BN1 9PX UK

**Keywords:** Breathlessness, Antidepressants, Economic evaluation, Off-label medication, Prescribing, Trial

## Abstract

**Background:**

Breathlessness is a prevalent, distressing symptom in advanced respiratory disease. Proven treatments are lacking. Mirtazapine, an antidepressant, is increasingly prescribed. The placebo-controlled BETTER-B trial did not find significant clinical benefit of mirtazapine for severe breathlessness, and patient experiences were mixed. Given mirtazapine’s low cost and wide availability, it is important to understand effects more broadly.

**Methods:**

We conducted a parallel cost-effectiveness analysis of mirtazapine versus placebo. BETTER-B recruited 225 adults with chronic obstructive pulmonary disease and/or Interstitial Lung Diseases in seven countries between 2021 and 2023. We calculated quality-adjusted life years (QALYs) using EuroQoL EQ-5D-5 L and national value sets, and combined self-reported healthcare and informal care frequencies with unit costs. Primary trial endpoint was at day 56, with sensitivity analysis at day 180 (trial exit).

**Findings:**

In primary analysis mirtazapine reduced QALYs (-0.006 (95% confidence interval (CI): -0.012 to -0.001)) and was associated with higher total costs (+€231 (95% CI: -218 to + 680)). For willingness-to-pay thresholds of €20,000-€40,000 per QALY, mirtazapine had a 1%-2% likelihood of being cost-effective. These findings were substantively unaffected by sensitivity analyses to timeframe, perspective, outcome measurement, and modelling strategy.

**Interpretation:**

Repurposing mirtazapine to treat severe breathlessness negatively impacted patient outcomes while being associated with higher formal and informal costs, and should be discouraged. Off-label prescribing of repurposed medicines without robust evidence risks unnecessary strain on healthcare systems and families. Economic evaluation within testing of repurposed medicines is important. Parallel cost-effectiveness analyses can deliver high-value information even when the efficacy trial finds no effect.

**Supplementary Information:**

The online version contains supplementary material available at 10.1186/s12913-025-13605-9.

## Research in context

### Evidence before this study

Burden-of-disease studies show that breathlessness is a prevalent, distressing symptom in advanced respiratory disease, straining patients, families and health systems. High-quality evidence reviews including Cochrane reviews have identified no proven treatments, once treatments to alleviate the disease are no longer effective and breathlessness is severe. Case and feasibility studies had indicated therapeutic potential for mirtazapine to modulate respiratory function, and a survey of clinicians identified increasing prescribing of mirtazapine. The multinational, placebo-controlled BETTER-B trial found no significant clinical benefit of mirtazapine. There has been no prior parallel economic evaluation of mirtazapine for breathlessness.

### Added value of this study

We identified a significant negative effect on patient quality of life from treating breathlessness using mirtazapine. Given higher formal and informal costs for those taking mirtazapine, the probability that this is a cost-effective strategy is very low. Our results illustrate more widely the dangers of off-label prescribing without rigorous research evidence.

### Implications of all the available evidence

Mirtazapine should not be prescribed for treating breathlessness. Off-label medications should be thoroughly assessed on a wide range of outcomes prior to repurposing. Economic evaluation is a valuable component of such assessment, providing a fuller picture and identifying dynamics not observed in analysis of a single clinical endpoint.

## Introduction

### Background

Respiratory diseases, including chronic obstructive pulmonary disease (COPD) and interstitial lung diseases (ILD), are the third most common cause of death in Europe [1]. Over 450 million people worldwide live with respiratory diseases [2]. Breathlessness is one of the most widespread symptoms in chronic respiratory disease, increases in severity with disease progression, is among the leading causes of morbidity among older people, causes distress to patients and families, and imposes significant burdens on formal healthcare systems, unpaid family care networks and the wider economy [3]. There is a lack of proven treatments for chronic and refractory breathlessness, once treatments to alleviate the disease are no longer effective and breathlessness is severe [4].

Mirtazapine is an antidepressant that is widely available at low cost [5]. BETTER-B was a research programme on breathlessness in advanced diseases [6]. Our review of case and feasibility studies had indicated therapeutic potential for mirtazapine to modulate respiratory function even in the absence of a mood disorder, [7] and a survey of respiratory and palliative care doctors in 13 European countries found prevalent off-label prescribing of anti-depressants and anti-anxiety drugs to alleviate breathlessness [8]. The BETTER-B programme included an international, multicentre, phase III, parallel-group, double-blind, randomised, placebo-controlled trial of mirtazapine, a widely-prescribed anti-depressant for breathlessness (trial registration: ISRCTN10487976, ISRCTN15751764 (Australia/New Zealand) EudraCT 2019-002001-21). The trial found no effect on the primary clinical endpoint of patient-reported *worst breathlessness* measured on a numerical rating scale at day 56 post-treatment start [9].

### Rationale and aims

Given mirtazapine’s low cost and wide availability, it is important to consider effects of repurposing more broadly. Although the BETTER-B trial did not show a clinical benefit from mirtazapine, clinicians may continue to prescribe off-label in the absence of proven treatments [10]. Furthermore, qualitative evidence from BETTER-B participants was mixed with some reporting benefits and others adverse events [9]. 

A parallel economic evaluation of clinical trial data can measure and integrate treatment effects across multiple domains, including health-related quality of life (HRQoL), formal care costs and informal care. Given the high reliance of people with severe breathlessness and respiratory disease on formal healthcare and unpaid family care, [11] small shifts in service use could raise or lower societal costs, regardless of clinical impact. Understanding these shifts is crucial, as they carry significant policy implications for European health systems.

Our aim was to evaluate and report the cost-effectiveness of mirtazapine compared to placebo for people with COPD and/or ILD in the UK, Germany, Ireland, Italy, Poland, Australia and New Zealand to the primary clinical endpoint (56 days) and trial exit (180 days).

## Methods

### Overview of the trial and study population

Full details of the BETTER-B study are available elsewhere (trial registration: ISRCTN10487976; registered 19/11/2019) [9]. Participants were recruited across 16 centres across the UK, Germany, Italy, Ireland, Poland, New Zealand and Australia. Eligible participants were aged 18 years or over, with COPD and/or ILD, grade 3 or 4 of the modified Medical Research Council (mMRC) breathlessness scale, [12] stable for the previous two weeks and on optimal treatment for reversible causes of breathlessness as judged by the referring clinician and according to best clinical guidance. Exclusion criteria included existing antidepressant use or other serotonergic active substances (e.g. linezolid, St John’s wort), known contraindication to mirtazapine, or Australia-modified Karnofsky Performance Scale ≤ 40 (= in bed more than 50% of the time or more disabled) due to the likely shorter prognosis and inability to complete the trial over 56 days.

The trial protocol, the written informed consent form, and other materials related to the participants were approved by the ethics committees at all sites. The trial was funded by the European Commission’s Horizon 2020 programme and the National Health and Medical Research Council (NHMRC) in Australia, and was performed in accordance with the Declaration of Helsinki and the Good Clinical Practice guidelines.

### Economic evaluation framework

#### Health economics analysis plan (HEAP)

A health economics analysis plan was developed and submitted to the European Commission, in 2021. We report our methods and results consistent with the Consolidated Health Economic Evaluation Reporting Standards 2022 (CHEERS2022) Statement (Appendix 1).

#### Perspective, time horizon and discount rate

In primary analysis, we report costs combining the formal health and social care use and unpaid family caregiving perspectives. This choice was made given the decision-making context for a repurposed medication already widely prescribed at low cost, where the relevant audience is not a single payer authority (such as NICE in the UK) but rather a wide range of stakeholders including prescribing clinicians. We also based the choice on investigator experience, the pivotal role of unpaid family carers in supporting those with life-limiting illness, and in particular the risk that reduced utilisation of formal care (e.g. fewer or shorter hospital admissions) may result in higher family care burden [11]. 

The primary endpoint was day 56 after randomisation and the trial endpoint was day 180, consistent with the clinical trial. In all analyses we report outcomes for patients. No discount rate was used given that the trial entry and outcomes were within a year. All choices were made at the trial outset.

#### Selection, measurement and valuation of outcomes

We measured participant HRQoL using the EuroQoL EQ-5D-5 L, since this is the gold standard for calculating quality-adjusted life years (QALYs) for use in health economic evaluation and particularly pharmacoeconomics. For six of the countries we combined responses with national value sets for attaching utility weights to EQ-5D-5 L scores [13–18]. For the UK, our original analysis plan had specified using a 5 L value set that is no longer recommended under local guidance; we therefore employed the recommended mapping model that translates 5 L responses to the 3 L value set [19]. Data were collected at baseline, day 28, day 56 and day 180. We calculated individual-level QALYs to day 56 (primary endpoint) and day 180 (trial endpoint), adjusting for timeframe using linear interpolation.

#### Selection, measurement and valuation of resources and costs

We developed a bespoke questionnaire (Client Services Receipt Inventory (CSRI)) for collecting frequency of relevant health, social and family care (Appendix 2). We developed the CSRI first in English among the consortium, then adapted to other local languages using forwards and backwards translation. We asked about formal care categories including hospital care (emergency department, inpatient admissions, outpatient appointments), community-based services (e.g., primary care physician, public health/community nursing, allied health), residential and social care, and equipment. We asked about informal care as assistance with basic and instrumental activities of daily living. Data were collected at baseline (prior three months’ healthcare use), day 28 (prior month), day 56 (prior month) and day 180 (prior three months). In primary analysis to day 56, the entire time period was covered by questionnaire responses. To estimate total costs to day 180, we addressed the unobserved period between day 57 and day 90 using linear interpolation.

We estimated costs by combining reported frequency in the CSRI with unit costs for each service. We aimed to collect country-specific unit costs for all services. We established a near-complete database of costs for the UK, using a combination of Personal Social Services Research Unit (PSSRU) database and National Health Service (NHS) tariffs. We established a near-complete database of costs for Ireland, using a combination of published databases and reimbursement data. We identified some unit costs for Germany, Italy, Poland and Australia, and none for New Zealand. In primary analysis we used national unit costs where available, and otherwise imputed the cost from available unit costs in other participating countries using relative health prices (Appendix 3). Where unit costs were available in different years, we adjusted to 2022 (the year when 70%< participants were recruited) using national health consumer price index (CPI), and then (for the UK and Australia) to Euros (€) using purchasing power parity (PPP). The cost of the intervention was identified using the study running costs and was approximately €0.45 per treatment group participant per day.

We describe baseline costs monthly and then estimated effect on costs for the relevant timeframe (56 days in primary analysis and selected sensitivity analyses, 180 days in selected sensitivity analyses).

### Statistical methods

#### Missing data due to attrition or mortality

For each cost data collection point, if a participant answered any CSRI question and left others blank then this blank was assumed to be zero. If the participant answered no CSRI questions then their utilisation data were deemed missing at random. Using this method there was no missing cost data at baseline; we imputed missing cost data among participants at follow-up using the same approach as living non-participants. For EQ-5D-5L at baseline, we predicted HRQoL score in a cross-sectional ordinary least squares regression where predictors were age, sex, country and those EQ-5D-5L domains with non-missingness, and we imputed this predicted value as HRQoL at baseline. We imputed missing HRQoL data among participants at follow-up using the same approach as living non-participants.

To account for missing data after baseline, we followed the approach of the main trial [9]. We estimated missing outcome variables using multiple imputation by chained equations (MICE; 50 imputations), assuming missing data were missing at random (MAR), [20] using as independent predictors age, sex, a binary multimorbidity variable (= 1 if Charlson comorbidity score of 3 or more) and a variable measuring outcome at baseline [21, 22]. For baseline costs, we log-transformed the predictor to increase normality of the distributed residuals. For baseline outcomes, we used the HRQoL score combining Eq. 5D5L responses with national value sets for utility. Mortality and date of death were identified as a part of the main trial. Where a participant’s death was reported between two data collection points, their costs and QALYs had to be imputed; we adjusted all such outcomes for the relevant time period using linear interpolation (e.g. if a death occurred midway through the period, we multiplied the imputed costs and outcomes by 0.5).

#### Analytics and sampling uncertainty

To account for differences arising from random variation in the sample, [23] we examined the treatment and control groups at baseline on important individual-level variables hypothesised as potentially impacting outcomes. Based on those results and following the main trial approach, we used the same predictors in treatment effect estimation as we had used in multiple imputation.

To account for skewed costs, [23] we modelled each cost outcome using a generalised linear model (GLM) with a gamma distribution and a log link, selected jointly after comparison with other distributions (Gaussian, Poisson) and links (Power), according to Akaike and Bayesian information criteria, using our selected predictors [23]. To account for the hierarchical structure of international trial data, and the concomitant risk of between-location variability biasing results, [24] we used multi-level GLMs for costs, clustering individual-level observations by country with random effects [25]. In primary analysis, we estimated treatment effects on costs at 56 days and on HRQoL at 56 days in separate regressions, using non-parametric bootstrapping with 1000 replications in each of the 50 MICE-generated datasets. We then combined these 50,000 bootstrapped estimates to calculate our main results: incremental cost-effectiveness ratio (ICER, calculated as the difference in costs between arms divided by the difference in QALYs between arms) and the associated probability that the treatment is cost-effective for willingness to pay in the range €20,000-€40,000 per QALY. Of our participating countries, the UK is the most explicit in setting a threshold between £20,000 and £30,000 (approximately €23,000 to €35,000) per QALY [26, 27]. While both average threshold and plausible ranges naturally differ across countries, health economics research and policy guidance for other countries in BETTER-B identify or estimate the implied threshold around this range [28–33]. 

Primary analyses were repeated in five sensitivity analyses: (1) perspective (using formal costs only in the outcome of interest to day 56), (2) timeframe (analysing data to trial exit at day 180), (3) unit cost estimation (using one set of unit costs, adjusted for other countries using relative prices), (4) QALY estimation (using one national value set, Italy, the country which had most participants among countries with a settled consensus on QALY estimation using Eq. 5D5L); and (5) modelling approach (using seemingly unrelated regressions instead of separate regressions) [23]. 

Unit costs were entered and adjusted for year and currency in Microsoft Excel. All other data processing and analyses were performed in Stata (version 17). In primary analysis, regressions were run using Stata programmes and author-written code, combining non-parametric bootstrapped estimates of treatment effect on costs and outcomes using the -*bsceaprogs.do*- set of programmes by Glick et al. [34] Sensitivity analyses using seemingly unrelated regressions were conducted using the code provided by Mutubuki et al. [23].

#### Characterising heterogeneity and distributional effects

For this article we report treatment effect estimates for the whole sample, and input parameters (costs and QALYs) by country (Appendix 5). We do not report formal sub-sample analyses.

#### Approach to engagement with patients and others affected by the study

A Patient and Public Involvement and Engagement (PPIE) group comprising patients and family carers affected by breathlessness was involved in study design, protocol development, trial monitoring and delivery. A patient-led organisation, The European Lung Foundation, was involved at all stages of the project. In addition to our trial steering and data quality and safety committees, an ethics advisory board oversaw the entire BETTER-B programme.

## Results

### Study parameters

The trial sample is presented on baseline factors in Table 1. Two hundred and twenty-five participants were randomised to either mirtazapine (*n* = 113) or placebo (*n* = 112). The average age of participants was 72.2 years, and 65.8% (*n* = 148) were male. More than three quarters of the sample were recruited in three of the seven countries – the UK (41.8%), Italy (18.2%) and Germany (16.0%). Mean HRQoL score at baseline (where 1 equals full health) was 0.61 (standard deviation: 0.24); 0.62 mirtazapine, 0.61 placebo.

**Table 1:** Baseline characteristics

	Mirtazapine (*n* = 113)	Placebo (*n* = 112)	Total (*n* = 225)
**Age (years)**			
Mean (s.d.)	72.8 (8.91)	71.7 (8.95)	72.2 (8.93)
**Gender**			
Male	73 (64.6%)	75 (67.0%)	148 (65.8%)
Female	40 (35.4%)	37 (33.0%)	77 (34.2%)
**Country of recruitment**			
UK	47 (41.6%)	47 (42.0%)	94 (41.8%)
Ireland	9 (8.0%)	10 (8.9%)	19 (8.4%)
Italy	20 (17.7%)	21 (18.8%)	41 (18.2%)
Germany	18 (15.9%)	18 (16.1%)	36 (16.0%)
Poland	10 (8.8%)	5 (4.5%)	15 (6.7%)
Australia	4 (3.5%)	6 (5.4%)	10 (4.4%)
New Zealand	5 (4.4%)	5 (4.5%)	10 (4.4%)
**Primary diagnosis**			
COPD	63 (55.8%)	61 (54.5%)	124 (55.1%)
ILD	50 (44.2%)	51 (45.5%)	101 (44.9%)
**Taking opioids**			
Yes	19 (16.8%)	17 (15.2%)	36 (16.0%)
No	94 (83.2%)	95 (84.8%)	189 (84.0%)
mMRC grade*			
Grade 3	75 (66.4%)	74 (66.1%)	149 (66.2%)
Grade 4	38 (33.6%)	38 (33.9%)	76 (33.8%)
**Charlson index summary score**			
Mean (s.d.)	1.8 (1.30)	1.6 (1.14)	1.7 (1.23)
**HRQoL**			
Mean (s.d.)	0.62 (0.23)	0.61 (0.24)	0.61 (0.24)
**Household – Living with**			
Alone	34 (30.1%)	28 (25.0%)	62 (27.7%)
Spouse/partner	56 (49.6%)	57 (50.9%)	113 (50.5%)
Children	3 (2.7%)	7 (6.3%)	10 (4.5%)
Friends	1 (0.9%)	0 (0%)	1 (0.5%)
Spouse/partner and Children	10 (8.9%)	14 (12.5%)	24 (10.7%)
Others	8 (7.1%)	6 (5.4%)	14 (6.3%)
**Informal care**			
Hours per week	32.9 (59.1)	17.9 (24.4)	25.7 (46.2)

Further details on baseline characteristics are provided in [16]

*mMRC: Modified Medical Research Council scale assessing baseline functional disability due to dyspnoea [20]. HRQoL: health-related quality of life where 1 = full health combining Eq. 5D5L and national value sets

Attrition to day 56 is presented in Table 2 and details of adherence for cost and HRQoL questionnaires specifically are provided in Appendix 5. Of the 225 participants at baseline, 81.2% (*n* = 181) participated to the primary endpoint. Of these 181, 178 (98.0%) had non-missing cost data and 173 (95.6%) had non-missing HRQoL data. At trial exit, of 159 participants who completed the primary clinical endpoint, 148 (93.1%) and 144 (90.6%) completed cost and HRQoL questionnaires respectively.

**Table 2:** Attrition at primary endpoint, day 56

	Mirtazapine (*n* = 113)	Placebo (*n* = 110)	Total (*n* = 223)
**Day 56**			
Primary clinical endpoint collected	88 (77.9%)	93 (84.5%)	181 (81.2%)
Missed Day 56 questionnaire, stayed in study	2 (1.8%)	3 (2.7%)	5 (2.2%)
ADD (attrition due to death)	3 (2.7%)	1 (0.9%)	4 (1.8%)
ADI (attrition due to illness)	12 (10.6%)	6 (5.5%)	18 (8.1%)
AAR (attrition at random)	8 (7.1%)	7 (6.4%)	15 (6.7%)

ADD = attrition due to death, ADI = attrition due to illness, AAR = attrition at random [39]. For further details of study adherence, see main clinical paper [9]

**Fig. 1:**
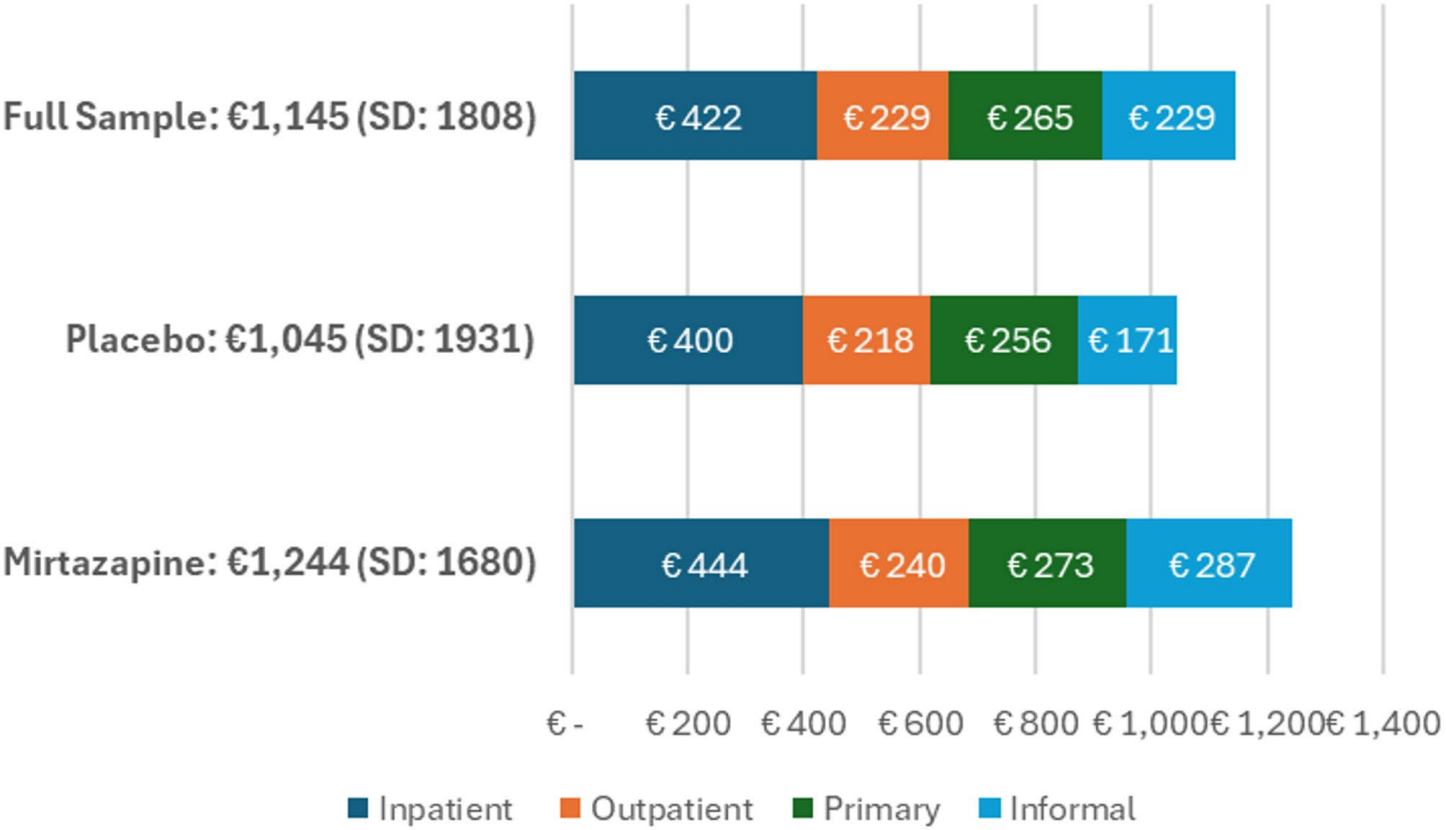
Mean monthly costs at baseline

Mean monthly costs at baseline are presented by study arm in Fig. 1. Mean costs were €1,145 (€1,244 mirtazapine, €1,045 placebo). Inpatient costs were the most substantial component (€422, 37%), with the remainder spread fairly evenly between outpatient (€229, 20%), primary and community care (€265, 23%) and informal care (€229, 20%). Mean monthly baseline costs were €201 higher in the treatment group, more than half of which was accounted for by difference in informal care.

### Summary of main results

**Fig. 2:**
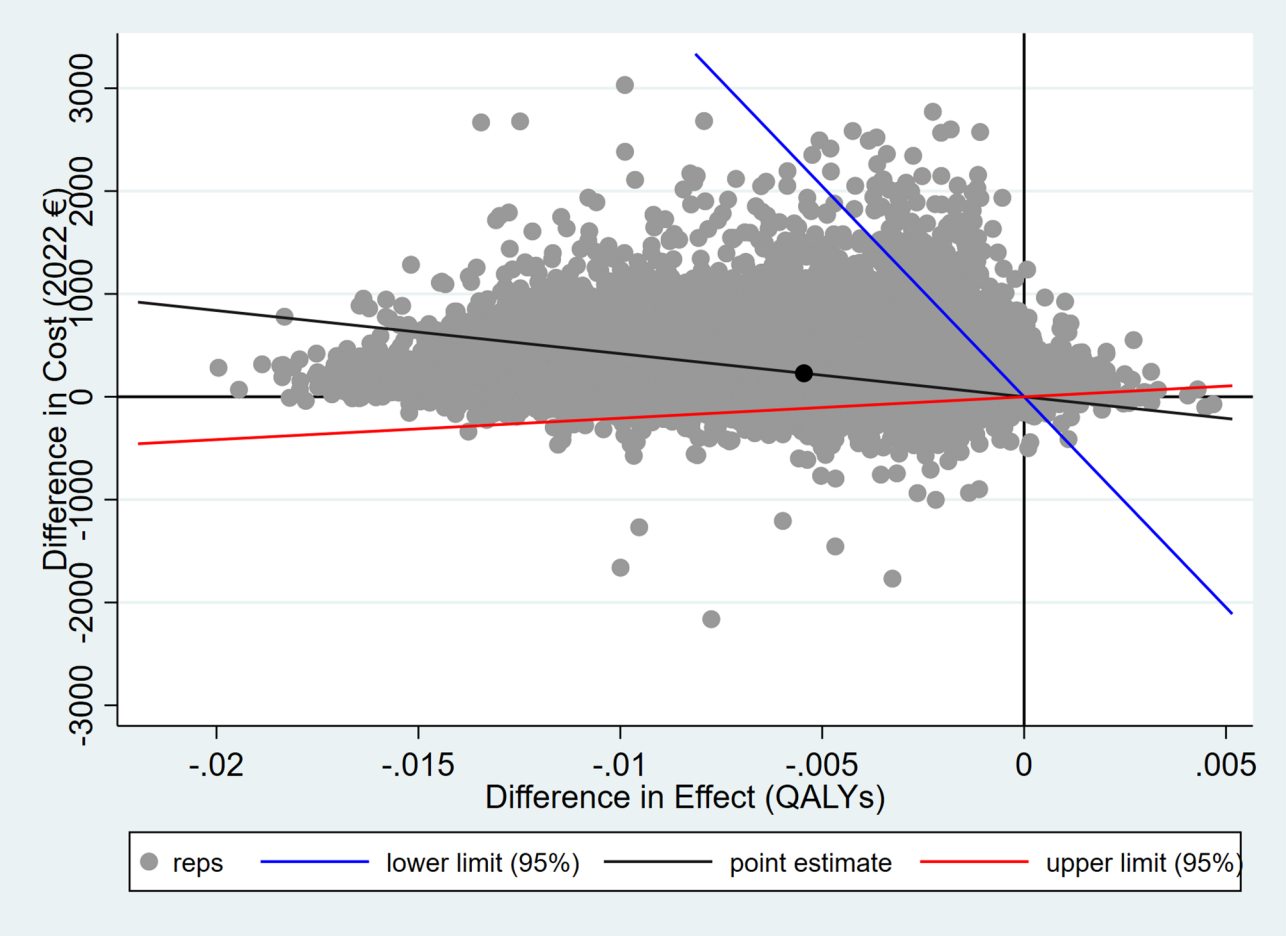
Cost-effectiveness plane: mirtazapine versus placebo, primary analysis to 56 days

The bootstrapped treatment effect estimates for our primary analyses are presented in Table 3, and the cost-effectiveness plane for these results is presented in Fig. 2.

**Table 3:** Bootstrapped treatment effect estimates, primary and sensitivity analyses

	Incremental cost (€, 95% CI)	Incremental QALYs (95% CI)	ICER (€ per QALY, 95% CI)	*p*_€20,000_	*p*_€30,000_	*p*_€40,000_
*Primary analysis*						
To day 56, formal + informal costs, separate regressions	231 (-218 to 680)	-0.006 (-0.012 to -0.001)	-37,903 (-385,000 to 16,600)	2%	1%	1%
*Sensitivity analyses*						
1 Perspective: formal costs only	116 (-82 to 314)	n/a	-18,989 (-173,009 to 1,374)	< 0.5%	< 0.5%	< 0.5%
2 Timeframe: to day 180	548 (-1318 to 2415)	-0.012 (-0.035 to 0.010)	-44,356 (n/d)	13%	12%	11%
3 Outcome measurement: unit costs	291 (-208 to 790)	n/a	-48,500 (-455,781 to 7,042)	2%	2%	1%
4 Outcome measurement: QALYs	n/a	-0.006 (-0.012 to -0.001)	-39,063 (-845,105 to 1,490)	1%	1%	1%
5 Modelling approach: SUR method	812 (-905 to 2457)	-0.004 (-0.012 to 0.004)	-209,514 (n/d)	16%	15%	14%

Incremental estimates derived from 50,000 regression outputs (1000 bootstrap replications x 50 MICE imputations). CI = confidence interval. ICER = incremental cost-effectiveness ratio. p_€*y*_= probability cost-effective if willing to pay €*y* per QALY. SUR = seemingly unrelated regressions. n/a: incremental effects estimated in primary analysis. n/d: 95% CI is not defined

Compared to placebo after 56 days in primary analysis, mirtazapine was associated with higher total costs (+€231, 95% confidence interval (CI): -218 to + 680), and inferior QALYs (-0.006, 95%CI: -0.012 to -0.001). The placebo therefore dominates the treatment at the mean (mean estimated ICER of -€37,903 per QALY (95%CI: -385,000 to 16,600)), and there is a probability of willingness to pay for treatment at €20,000-€40,000 per QALY of 1–2%.

### Effect of uncertainty

Primary analyses were repeated in four sensitivity analyses: (1) perspective (using formal costs only in the outcome of interest to day 56), (2) timeframe (analysing data to trial exit at day 180), (3) unit cost estimation (using one set of unit costs, adjusted for other countries using relative prices), (4) QALY estimation (using one national value set, Italy, the country which had most participants among countries with a settled consensus on QALY estimation using EQ-5D-5L); and (5) modelling approach (using seemingly unrelated regressions instead of separate regressions). These results are presented in Table 3. Mean estimated ICER was negative in each of the four analyses, indicating the dominance of placebo over treatment. Restricting the cost perspective to formal costs increased the dominance of placebo compared to the primary results, delivering a probability of cost-effectiveness of less than 1% for all specified thresholds. Alternative approaches to estimating unit costs and QALYs delivered results very closely aligned with the primary analysis. Sensitivity to timeframe and modelling approach was somewhat higher. Both sets of results had higher levels of uncertainty, and consequently somewhat higher probability of mirtazapine’s cost-effectiveness. Nevertheless, the interpretation that mirtazapine was associated with higher costs and lower quality of life, and as such has a very low probability of cost-effectiveness, holds across all analyses.

## Discussion

### Key results

This parallel economic evaluation within the BETTER-B trial comparing mirtazapine to placebo for treating severe breathlessness in respiratory diseases found that mirtazapine negatively impacted patient outcomes while being associated with higher formal and informal care costs. Consequently, placebo dominated treatment at the mean, and the probability that mirtazapine is cost-effective was very low. This interpretation was substantively unaffected by sensitivity analyses to timeframe, cost perspective, unit cost inputs and modelling strategy.

Our results have at least three important implications for current practice and policy, and for future research. First, mirtazapine should not be widely used to treat chronic breathlessness. Our main trial reported a null finding on the primary clinical outcome worst breathlessness, [9] which may not deter prescribing of a low-cost, widely-available medication in this challenging clinical context [10]. The wider scope of economic evaluation captures hitherto unidentified patient adverse outcomes as well as an association with higher costs, which in any individual patient in advanced illness, might be attributed to worsening disease, rather than the intervention itself. Our results make an altogether stronger case against this intervention in this population. It is unusual for trials in seriously-ill populations to include an economic evaluation, [35] although a similar example of economic evidence strengthening clinical evidence in this field was seen in a recent trial of sustained-release morphine for refractory breathlessness in COPD [36]. 

Second, our results demonstrate how off-label prescribing without rigorous research evidence may harm patients and increase burdens on systems and families. A survey conducted in 13 European countries prior to the BETTER-B trial found that respiratory medicine and palliative care doctors often resort to off-label prescribing of anti-depressants and anti-anxiety drugs to alleviate breathlessness [8]. The increasing prevalence of mirtazapine prescribing based on weak evidence is consistent with prior experience in breathlessness with benzodiazepines [10]. International evidence shows increasing use of off-label antidepressants not only for breathlessness but also pain, insomnia and other symptoms [37]. While it is understandable that clinicians may be willing to try low-cost medications, absent proven alternatives for patients in distress, our results caution strongly against this temptation. While there is ongoing debate about the benefits and challenges of repurposing existing drugs, [38] our findings emphasise the need for rigorous testing of repurposed drugs to ensure they are cost-effective and safe.

Third, economic evaluation is a vital component of such testing. In the development and testing of new medications, economic evaluation often occurs after clinical efficacy has been proven in order to demonstrate value to a specific payer (e.g. NICE in the UK, HIQA in Ireland, etc.). This process is reflected in methodological and reporting practices that privilege the decision-making criteria of a specific policymaking authority. Such practices have low relevance for repurposed medications already widely prescribed at low cost. In this case the relevant audience is not a single authority but rather a wide range of stakeholders encompassing not only policymakers but also respiratory clinical teams, pharmacists, patients and families, patient groups, and researchers. Parallel economic evaluation within a trial of repurposed medications is the most efficient way to generate high-quality evidence to this end. Possibly some methodological and reporting guidance in trials and economic evaluation specific to repurposed medicines are warranted in order to promote understanding of evidence consistent with the decision-making context. For example, exploring not only the system perspective but also the impact on family caregivers’ time and well-being may be valuable where clinicians are prescribing superficially low-cost drugs on an assumption that there is little possible downside. Beyond the realm of repurposed medicines, our study reinforces a simple but important point that parallel cost-effectiveness analyses can deliver high-value information even when the efficacy trial finds no effect. As others have noted, [39] there is scope to improve understanding of this issue among researchers and clinicians.

### Strengths

BETTER-B represents one of the largest phase III trials performed in severe breathlessness and an opportunity to evaluate if mirtazapine represents a cost-effective treatment for health systems in treating the large and growing population health burden of chronic respiratory diseases, especially in severe disease when hospital admissions are common and strain on informal carers is high [11]. In addition to the trial size and rigour, our paper’s strengths include the flexible approach to perspective, measuring how repurposed medications may have negative not only for health systems but also for family caregivers, who are typically out of the scope of a basic economic evaluation. Furthermore, studies that have managed to collate unit costs on multiple countries are rare. This paper tackled the significant challenges undertaking multi-country CEAs using best-practice methods of country-specific unit costs, and underlines the importance of large-scale funded projects taking a systematic, purposive approach to calculating international unit costs.

### Limitations

Recruitment challenges that may impact interpretation of results included the COVID-19 pandemic, and the ineligibility of 25% of screened candidates as they were already receiving antidepressants. Heterogeneity of response among individuals, in the nature of respiratory of diseases and national-level approaches to breathlessness care may also have affected results. Generalisability to overall population of people with chronic breathlessness in different countries is unclear. The trial was underpowered compared to the original protocol but the main clinical paper concluded that full recruitment was unlikely to alter key conclusions. Power was also not obviously a concern for this cost-effectiveness analysis, which found a statistically significant effect on patient outcomes in primary analysis. The most important difference between groups on baseline characteristics was on prior costs, and in particular informal costs. We aimed to manage that difference by including baseline costs as a predictor both in multiple imputation and in estimating treatment effects, and by conducting sensitivity analysis by cost perspective.

There are a number of well-known challenges in economic evaluation within international trials for which no standardised guidance exists, in particular how to manage differences between countries in prices and patterns of care [40]. We aimed to manage heterogeneous patterns of care, resource use and response to treatment across countries in analysis and sensitivity analysis. Other generic limitations include recall bias in CSRI responses, reliability of family care reporting and sensitivity of EQ-5D-5 L to disease-specific contexts. While we did include informal costs, we examined outcomes only from the patient perspective; we will examine caregiver outcomes in future work. We did not perform sub-sample analyses within our data; in the context of prior evidence that there is significant treatment effect heterogeneity among people with life-limiting illness, [41] we will further explore these dynamics in future work.

## Conclusion

Mirtazapine had a significant negative effect on patient outcomes in severe breathlessness, offers poor value care compared to placebo and should not be widely prescribed. Repurposing of medication with adverse effects for patients, families and the health system is not only a concern for this specific patient group but also illustrates the risks of off-label prescribing without rigorous research evidence. Economic evaluation is a key component of such evidence, measuring effects on outcomes beyond primary clinical endpoints and ensuring that those effects are understood in a decision-making context where prescribing a medication with limited or equivocal benefits may erroneously appear a low-risk strategy.

## Supplementary Information

Below is the link to the electronic supplementary material.

Supplementary Material 1

Supplementary Material 2

Supplementary Material 3

Supplementary Material 4

Supplementary Material 5

Supplementary Material 6

## Data Availability

Data collected for the study, including de-identified participant data, data dictionary, and additional related documents, will be made available to others upon request to better-b@kcl.ac.uk, according to the King’s College London data sharing policy and in accordance with WHO statement on public disclosure of clinical trial results.
