# Supplementary material for: The cost of drug repurposing: parallel economic evaluation of mirtazapine for severe breathlessness in the multinational BETTER-B trial
Source: BMC Health Serv Res. 2025 Nov 4;25:1442. doi: 10.1186/s12913-025-13605-9 (PMC12584416; doi:10.1186/s12913-025-13605-9)
Supplement: Supplementary file 1 — Supplementary Material 1 [file 12913_2025_13605_MOESM1_ESM.pdf]

# Appendix 1: CHEERS checklist

## Title

1 Identify the study as an economic evaluation and specify the interventions being compared. TITLE PAGE

## Abstract

2 Provide a structured summary that highlights context, key methods, results, and alternative analyses. marked 'Abstract'

## Introduction

*Background and objectives*

3 Give the context for the study, the study question, and its practical relevance for decision making in policy or practice. Page 5 of 19

## Methods

*Health economic analysis plan*

4 Indicate whether a health economic analysis plan was developed and where available. Page 6 of 19

*Study population*

5 Describe characteristics of the study population (such as age range, demographics, socioeconomic, or clinical characteristics). Page 6 of 19

*Setting and location*

6 Provide relevant contextual information that may influence findings. Page 6 of 19

*Comparators*

7 Describe the interventions or strategies being compared and why chosen. Page 6 of 19

*Perspective*

8 State the perspective(s) adopted by the study and why chosen. Page 6 of 19

*Time horizon*

9 State the time horizon for the study and why appropriate. Page 6 of 19

*Discount rate*

10 Report the discount rate(s) and reason chosen. Page 6 of 19

*Selection of outcomes*

11 Describe what outcomes were used as the measure(s) of benefit(s) and harm(s). Page 7 of 19

*Measurement of outcomes*

12 Describe how outcomes used to capture benefit(s) and harm(s) were measured. Page 7 of 19

Valuation of outcomes

13 Describe the population and methods used to measure and value outcomes. Page 7 of 19

*Measurement and valuation of resources and costs*

14 Describe how costs were valued. Page 7 of 19 plus Appendices 2 and 3

*Currency, price date, and conversion*

15 Report the dates of the estimated resource quantities and unit costs, plus the currency and year of conversion. Page 7 of 19 plus Appendix 3

*Rationale and description of model*

16 If modelling is used, describe in detail and why used. Report if the model is publicly available and where it can be accessed. N/A

*Analytics and assumptions*

17 Describe any methods for analysing or statistically transforming data, any extrapolation methods, and approaches for validating any model used. Page 8 of 19

*Characterising heterogeneity*

18 Describe any methods used for estimating how the results of the study vary for subgroups. Page 8 of 19

*Characterising distributional effects*

19 Describe how impacts are distributed across different individuals or adjustments made to reflect priority populations. Page 8 of 19

*Characterising uncertainty*

20 Describe methods to characterise any sources of uncertainty in the analysis. Page 9 of 19

*Approach to engagement with patients and others affected by the study*

21 Describe any approaches to engage patients or service recipients, the general public, communities, or stakeholders (such as clinicians or payers) in the design of the study. Page 9 of 19

## Results

*Study parameters*

22 Report all analytic inputs (such as values, ranges, references) including uncertainty or distributional assumptions. Page 10-11 of 19

*Summary of main results*

23 Report the mean values for the main categories of costs and outcomes of interest and summarise them in the most appropriate overall measure. Page 12-13 of 19

*Effect of uncertainty*

24 Describe how uncertainty about analytic judgments, inputs, or projections affect findings. Report the effect of choice of discount rate and time horizon, if applicable. Page 12-13 of 19 plus Appendices 4 and 5

*Effect of engagement with patients and others affected by the study*

25 Report on any difference patient/service recipient, general public, community, or stakeholder involvement made to the approach or findings of the study Page 9 of 19

## Discussion

*Study findings, limitations, generalisability, and current knowledge*

26 Report key findings, limitations, ethical or equity considerations not captured, and how these could affect patients, policy, or practice. Page 14 of 19

*Other relevant information*

Source of funding

27 Describe how the study was funded and any role of the funder in the identification, design, conduct, and reporting of the analysis Page 16 of 19

Conflicts of interest

28 Report authors conflicts of interest according to journal or International Committee of Medical Journal Editors requirements. Page 16 of 19
