# Supplementary material for: The cost of drug repurposing: parallel economic evaluation of mirtazapine for severe breathlessness in the multinational BETTER-B trial
Source: BMC Health Serv Res. 2025 Nov 4;25:1442. doi: 10.1186/s12913-025-13605-9 (PMC12584416; doi:10.1186/s12913-025-13605-9)
Supplement: Supplementary file 2 — Supplementary Material 2 [file 12913_2025_13605_MOESM2_ESM.pdf]

Appendix 2

Services and Care

This questionnaire is about your use of health and social care, and informal care (i.e. given by family members) **over the last 1 month**. Information provided here is used to calculate the costs of care.

Section 1: HOSPITAL/RESIDENTIAL SERVICES

In the last 1 month, have you **stayed** overnight in a hospital/other residential care setting?

Yes ☐ 1 No ☐ 0 **If you answered 'No', please go to section 2.**

If you answered 'Yes', please state the total number of attendances and the total number of days you stayed in any of the following settings during the last 1 month.

	Service	No. of times admitted	No. of days in total	How many (if any) of these days were spent in an intensive/critical care unit?	How many (if any) of these days were spent in a specialist rehabilitation ward/unit?
A.	Hospital inpatient ward				

	Service	No. of times admitted	No. of days in total
B.	Hospice		
C.	Nursing or Residential Home		
D.	Respite care setting		

Section 2: OUTPATIENT HOSPITAL SERVICES

Please respond with the number of times you have used each of the following emergency/outpatient services in the last 1 month. If you have not used a service, enter 0.

	Service	No. of visits
A.	Emergency ambulance service	
B.	Emergency Department<br>(sometimes called A&E department or Accident and Emergency)	
C.	Pulmonary rehabilitation	
D.	Specialist medicine outpatient visit (e.g. respiratory, cardiac, oncology)	
E.	Other hospital outpatient visit	
F.	Day hospital department (e.g., respiratory, cardiac, oncology)	

Section 3: COMMUNITY-BASED SERVICES

In the last 1 month, have you had any community-based appointments or consultations? This can include face-to-face, telephone or email consultations.

Yes ☐ 1 No ☐ 0 **If you answered 'No', please go to section 4.**

If you answered 'Yes', please state the number of times you have used any of the following community-based services over the last 1 month. Please also state the average contact time (in minutes) for each service you have used.

Service		Total number of contacts over the last 1 month	Average contact time/visit (minutes)	How many (if any) of these were out-of-hours contacts (e.g., at weekends, outside of normal office hours)?
A.	General Practitioner (GP) / Primary Care Physician			
B.	General practice nurse			
C.	District / Public Health / Community nurse			
D.	Physiotherapist			
E.	Occupational therapist			
F.	Speech & Language therapist			
G.	Dietician			
H.	Psychologist			
I.	Psychiatrist			
J.	Pulmonary rehabilitation (not including any reported in Section 2)			
K.	Specialist palliative care doctor / consultant (community-based consultation)			

L.	Palliative care nurse			
M.	Home palliative care / hospice service			
N.	Social worker			
O.	Home help / care worker / health care assistant			
P.	Dentist			
Q.	Optician / Optometrist			
R.	Other healthcare professionals in the community (please state)<br>1.<br>2.<br>3.			

Section 4: DIAGNOSTIC TESTS

In the last 1 month, have you had any investigations / diagnostic tests?

Yes ☐ 1 No ☐ 0 **If you answered 'No', please go to section 5.**

If you answered 'Yes', please fill in the investigations / diagnostic test you have received in the last 1 month.

Investigation / Diagnostic Test		Number in the last 1 month
A.	Full lung function	
B.	Chest x-ray	
C.	Echocardiogram	
D.	Electrocardiogram (ECG)	
E.	Blood gas test	
F.	Magnetic Resonance Image (MRI)	
G.	CT / CAT scan	
H.	Blood test	

Section 5: MEDICATION PROFILE

In the last 1 month, have you had any medication?

Yes ☐ 1 No ☐ 0 **If you answered 'No', please go to section 6.**

If you answered 'Yes', please fill in the medication you have received in the last 1 month.

**Name of drugs**		**Dosage (if known)**	**Dosage frequency**<br>1 = 3 times daily<br>2 = 2 times daily<br>3 = Once daily<br>4 = Weekly<br>5 = Every 2 weeks<br>6 = Monthly
A.			
B.			
C.			
D.			

Section 6: INFORMAL CARE

Please give details of any help you have received from **friends or family** members in the last 1 month **as a result of your illness.**

	Type of help	Average number of hours per week
A.	Personal care (e.g. washing, dressing, using the toilet, getting into bed)	
B.	Help with medical procedures (e.g. home oxygen or nebulisers)	
C.	Help inside/around the home (e.g. cooking, cleaning)	
D.	Help outside the home (e.g. shopping, transport)	
E.	Time spent 'on-call' i.e. needing someone to be with them even if they don't have specific needs for help	
F.	Other, if any (please state)<br>1.<br>2.<br>3.	

Section 7: EQUIPMENT

Please list below any **equipment** you have been using over the last 1 month.

Name of equipment		Yes/No
A.	Ambulatory oxygen (oxygen cylinders)	
B.	Long term oxygen therapy (oxygen concentrator)	
C.	Non-invasive ventilation (or CPAP):	
	Overnight	
	During the day	
	Both overnight & during the day	
D.	Walking aid (e.g., Rollator)	
E.	Wheelchair – manual	
F.	Wheelchair – electric	
G.	Feeding pump	
H.	Commode	
I.	Special bed	
J.	Bathroom or toilet adapted	
K.	Other equipment, if any (please state)<br>1.<br>2.<br>3.<br>Etc.	
