# Supplementary material for: The cost of drug repurposing: parallel economic evaluation of mirtazapine for severe breathlessness in the multinational BETTER-B trial
Source: BMC Health Serv Res. 2025 Nov 4;25:1442. doi: 10.1186/s12913-025-13605-9 (PMC12584416; doi:10.1186/s12913-025-13605-9)
Supplement: Supplementary file 3 — Supplementary Material 3 [file 12913_2025_13605_MOESM3_ESM.pdf]

# Appendix 3: Cost estimation: Unit cost database and calculation methods

## Overview

We collected frequency of healthcare use by BETTER-B participants using a bespoke Client Service Receipt Inventory (CSRI; see Appendix 2). To estimate costs associated with reported use we combined frequencies with unit costs and summed. The purpose of this appendix is to detail the identification of unit costs, the standardisation of unit costs to euros (€) for 2022, the year when the bulk of data were collected, and the imputation of unit costs where none could be identified for a given service in a given country. Finally, we detail how costs and frequencies were combined to generate our cost variables in analysis.

Two large-scale studies have recently examined the availability of unit costs for health and social care in Europe: the European ProgrammE in Costing, resource use measurement and outcome valuation for Use in multi-sectoral National and International health economic evaluAtions (PECUNIA)[1], and the European Healthcare and Social Cost Database (EU HCSCD).[2] Both have reported a lack of suitable unit costs in many European countries. Previously, the WHO-CHOICE programme has reported cost per inpatient bed day and per outpatient visit for many countries, although the data currently available were from 2008.[3]

Consequently, for the BETTER-B study we compiled a bespoke unit cost database for the relevant countries, and health and social care services, expecting that some unit costs would be unavailable and that unavailability would vary by country. In our original statistical analysis plan, we aimed to identify unit costs for each CSRI item in each of the five participating European countries. The additional study in Australia and New Zealand brought the total number of participating countries to seven.

For formal costs, there was 'duplication' of unit costs in some cases; that is, for a given service in a given country, we identified more than one unit cost. Given the scale and complexity compiling this database, and the variability in prevalence of such 'duplication' within and across countries, we did not retain multiple unit costs for a given service in a given country. Rather, we identified one unit cost per service per country, and then tested uncertainty around unit cost estimates for all unit costs in post hoc analysis (Sensitivity Analysis, Appendix 5). In any context with 'duplication', we prioritised national unit costs where available; if a regional (e.g. state-level) cost was identified but a national cost was not, we adopted the regional cost. Where 'duplication' occurred in different years, we took the unit cost closest to 2022; where multiple unit costs for the same service in the same year were

available from different sources, we took the unit cost from the source that contributed most unit costs to our study overall; where multiple unit costs for the same service in the same year were available from the same source, we took the median value.

For informal costs, the challenges specifying a unit cost have been extensively covered in the literature, and four main approaches identified: the opportunity cost approach, the replacement cost approach, contingent valuation, and conjoint analysis.[4] We used a combination of replacement and opportunity cost approaches, described in more detail below.

Since there is substantial missingness in formal unit costs, and no missingness in informal unit costs, we present these separately. The explanation of **formal** unit cost calculation is presented in three stages. In 3.1 we present country-by-country summary of formal unit costs identified. We include sources of unit costs, and the source of health consumer price index (CPI) used to adjust all identified costs to 2022 in the local currency. In 3.2 we summarise how units of service were standardised across countries, how national costs were converted into 2022 euros (€) for pooled analysis and how missing **formal** unit costs were imputed for primary analysis. In 3.3 we present the unit costs for **formal** care used in primary analysis. In 3.4 we present the **informal** care unit costing methods.

Further details on specific costs or references are available on reasonable request to the corresponding author.

## 3.1 Identified unit costs for **formal** care in participating countries

### 3.1.1 England

The UK has two mature repositories of unit costs - the Personnel Social Services Research Unit (PSSRU) database,[5] and the National Health Service (NHS).[6] Where neither database had a relevant unit cost, we examined sources recommended by PECUNIA and EU HCSCD, and we searched PubMed (MEDLINE) for recent economic evaluations that included an estimated unit cost. Local currency is GBP (£); we standardised costs to 2022 using the UK health CPI.[7]

*Table 1 Identified unit costs for the UK, 2022 £*

| | Unit cost (2022 £) | Per | Source |
|---|---|---|---|
| **Hospital** | | | |
| Hospital inpatient ward | £622 | Night | NHS[6] |
| Intensive/Critical care ward | £1,991 | Night | NHS[6] |
| Specialist rehab ward/unit | £586 | Night | PSSRU[5] |
| **Other residential** | | | |
| Hospice | £478 | Night | Round et al.[8] |

| | | | |
|---|---|---|---|
| Nursing or residential home | £165 | Night | PSSRU[5] |
| Respite care setting | £100 | Night | NHS[6] |
| **Outpatient hospital** | | | |
| Emergency ambulance | £272 | Visit | PSSRU[5] |
| Emergency dept (A&E) | £118 | Visit | Round et al.[8] |
| Pulmonary rehab | £195 | Visit | PSSRU[5] |
| Specialist (e.g., respiratory, cardiac, oncology) | £195 | Visit | PSSRU[5] |
| Other outpatient visit | £139 | Visit | PSSRU[5] |
| Day hospital (e.g., cardiac, oncology, respirator) | £777 | Visit | PSSRU[5] |
| **Community-based** | | | |
| General Practitioner/ Primary Care Physician | £41 | Visit | PSSRU[5] |
| General practice nurse | £39 | Hour | PSSRU[5] |
| District/Public health/ Community nurse | £39 | Hour | PSSRU[5] |
| Physiotherapist | £50 | Hour | PSSRU[5] |
| Occupational therapist | £50 | Hour | PSSRU[5] |
| Speech & language therapist | £50 | Hour | PSSRU[5] |
| Dietitian | £50 | Hour | PSSRU[5] |
| Psychologist | £50 | Hour | PSSRU[5] |
| Psychiatrist | £- | - | |
| Pulmonary rehab | £58 | Visit | NHS[6] |
| Specialist palliative care doctor/consultant | £- | - | |
| Palliative care nurse | £118 | Hour | NHS[6] |
| Home palliative care/hospice | £118 | Hour | NHS[6] |
| Social worker | £46 | Hour | PSSRU[5] |
| Home help/care worker/ health care assistant | £31 | Hour | PSSRU[5] |
| Dentist | £108 | Hour | PSSRU[5] |
| Optician/Optometrist | £100 | Hour | Hassiotis et al.[9] |
| **Diagnostic tests** | | | |
| Full lung function | £13 | Test | NIHR SoECAT Tariff[10] |
| Chest x-ray | £57 | Test | NIHR SoECAT Tariff[10] |
| Echocardiogram | £208 | Test | NIHR SoECAT Tariff[10] |
| Electrocardiogram (ECG) | £32 | Test | NIHR SoECAT Tariff[10] |
| Blood gas test | £30 | Test | NIHR SoECAT Tariff[10] |
| Magnetic Resonance Image (MRI) | £464 | Test | NIHR SoECAT Tariff[10] |
| CT/CAT Scan | £160 | Test | NIHR SoECAT Tariff[10] |
| Blood test | £30 | Test | NIHR SoECAT Tariff[10] |
| **Equipment** | | | |
| Ambulator oxygen (cylinders) | £91 | Treatment | NHS[6] |
| Long term oxygen therapy (oxygen concentrator) | £107 | Treatment | NHS[6] |
| Non-invasive ventilation (or CPAP) | £435 | Treatment | NHS[6] |
| Walking aid (e.g., Rollator) | £57 | Item | Hassiotis et al.[9] |
| Wheelchair - manual | £106 | Item | NHS[6] |
| Wheelchair - electric | £497 | Item | NHS[6] |
| Feeding pump | £- | - | - |
| Commode | £25 | Item | Hassiotis et al.[9] |
| Special bed | £- | - | - |

| | | | |
|---|---|---|---|
| Bathroom or toilet adapted | £596 | Item | PSSRU[5] |

### 3.1.2 Ireland

Members of the BETTER-B research team participated in a recent unit costing exercise for health and social care in Ireland.[11] Ireland was included in neither PECUNIA nor EU HCSCD. Other sources were identified using author knowledge and literature reviews. Local currency is EUR (€); we standardised costs to 2022 using the Irish health CPI.[12]

*Table 2 Identified unit costs for Ireland, 2022 €*

| | Unit cost (2022 €) | Per | Source |
|---|---|---|---|
| **Hospital** | | | |
| Hospital inpatient ward | € 2,024 | Night | Author calculation*[13] |
| Intensive/Critical care ward | € 3,151 | Night | Mastrogianni et al.[14] |
| Specialist rehab ward/unit | - | - | |
| **Other residential** | | | |
| Hospice | € 786 | Night | Personal communication |
| Nursing or residential home | € 148 | Night | Smith et al.[11] |
| Respite care setting | - | - | |
| **Outpatient hospital** | | | |
| Emergency ambulance | - | - | |
| Emergency dept (A&E) | € 321 | Visit | Keegan et al.[15] |
| Pulmonary rehab | € 190 | Visit | Carty et al.[16] |
| Specialist (e.g., respiratory, cardiac, oncology) | € 184 | Visit | Keegan et al.[15] |
| Other outpatient visit | € 184 | Visit | Keegan et al.[15] |
| Day hospital (e.g., cardiac, oncology, respirator) | € 841 | Visit | Healthcare Pricing Office[17] |
| **Community-based** | | | |
| General Practitioner/ Primary Care Physician | € 43 | Visit | Walsh et al.[18] |
| General practice nurse | € 17 | Visit | Walsh et al.[18] |
| District/Public health/ Community nurse | € 44 | Visit | Smith et al.[11] |
| Physiotherapist | € 34 | Visit | Smith et al.[11] |
| Occupational therapist | € 34 | Visit | Smith et al.[11] |
| Speech & language therapist | € 68 | Visit | Smith et al.[11] |
| Dietitian | € 34 | Visit | Smith et al.[11] |
| Psychologist | € 104 | Visit | Smith et al.[11] |
| Psychiatrist | € 231 | Visit | Brick et al.[19] |
| Pulmonary rehab | € 58 | Visit | Carty et al.[16] |
| Specialist palliative care doctor/consultant | € 121 | Visit | Brick et al.[19] |
| Palliative care nurse | € 45 | Visit | Brick et al.[19] |
| Home palliative care/hospice | € 111 | Visit | Brick et al.[19] |
| Social worker | € 30 | Visit | Brick et al.[19] |

* Healthcare Pricing Office ABF figures reflect the mean absorbed cost of an admission by DRG, including ICU admission. We combined these ABF data with published data on the unit cost and total bed days of ICU care to estimate the unit cost of an acute inpatient day *without* ICU.

| | | | |
|---|---|---|---|
| Home help/care worker/ health care assistant | € 35 | Visit | Smith et al.[11] |
| Dentist | € 95 | Visit | Smith et al.[20] |
| Optician/Optometrist | - | - | |
| **Diagnostic tests** | | | |
| Full lung function | - | - | |
| Chest x-ray | - | - | |
| Echocardiogram | - | - | |
| Electrocardiogram (ECG) | - | - | |
| Blood gas test | - | - | |
| Magnetic Resonance Image (MRI) | - | - | |
| CT/CAT Scan | - | - | |
| Blood test | - | - | |
| **Equipment** | | | |
| Ambulator oxygen (cylinders) | - | - | |
| Long term oxygen therapy (oxygen concentrator) | - | - | |
| Non-invasive ventilation (or CPAP) | - | - | |
| Walking aid (e.g., Rollator) | - | - | |
| Wheelchair - manual | - | - | |
| Wheelchair - electric | - | - | |
| Feeding pump | - | - | |
| Commode | - | - | |
| Special bed | €3,750 | Item | Author calculation[†] |
| Bathroom or toilet adapted | - | - | |

### 3.1.3 Germany

We were unable to identify a definitive unit cost database for Germany, which operates a federal healthcare system. Both PECUNIA and EU HCSCD estimated unit costs for Germany. We used results from PECUNIA and EU HCSCD, and to address extant gaps we searched PubMed (MEDLINE) for recent economic evaluations. Local currency is EUR (€); we standardised costs to 2022 using the OECD Health CPI for Germany.[21]

*Table 3 Identified unit costs for Germany, 2022 €*

| | Unit cost (2022 €) | Per | Source |
|---|---|---|---|
| **Hospital** | | | |
| Hospital inpatient ward | € 648 | Night | Bock et al.[22] |
| Intensive/Critical care ward | € 1,506 | Night | Bock et al.[22] |
| Specialist rehab ward/unit | - | - | |

[†] 'Special bed' was the one item in the CSRI for which no unit cost was identified in the academic or government literature for any country. We surveyed the website of three suppliers of medical beds for use in the home in Ireland, identified via Google. For the supplier with the widest range of products (12 beds; https://www.gomobility.ie/product-category/bedroom-aids/profiling-beds/), we took the median price.

| | | | |
|---|---|---|---|
| **Other residential** | | | |
| Hospice | - | - | |
| Nursing or residential home | € 146 | Night | PECUNIA[1] |
| Respite care setting | - | - | |
| **Outpatient hospital** | | | |
| Emergency ambulance | € 629 | Hour | Pöhlmann et al.[23] |
| Emergency dept (A&E) | € 135 | Visit | Pöhlmann et al.[23] |
| Pulmonary rehab | - | - | |
| Specialist (e.g., respiratory, cardiac, oncology) | € 74 | Visit | Pöhlmann et al.[23] |
| Other outpatient visit | - | - | |
| Day hospital (e.g., cardiac, oncology, respirator) | € 421 | Visit | Pöhlmann et al.[23] |
| **Community-based** | | | |
| General Practitioner/ Primary Care Physician | € 23 | Visit | PECUNIA[1] |
| General practice nurse | € 16 | Hour | Pöhlmann et al.[23] |
| District/Public health/ Community nurse | - | - | |
| Physiotherapist | € 19 | Visit | PECUNIA[1] |
| Occupational therapist | € 42 | Visit | Bock et al.[22] |
| Speech & language therapist | € 44 | Visit | Bock et al.[22] |
| Dietitian | € 34 | Visit | Pöhlmann et al.[23] |
| Psychologist | - | - | |
| Psychiatrist | € 50 | Visit | Bock et al.[22] |
| Pulmonary rehab | - | - | |
| Specialist palliative care doctor/consultant | - | - | |
| Palliative care nurse | - | - | |
| Home palliative care/hospice | - | - | |
| Social worker | - | - | |
| Home help/care worker/ health care assistant | - | - | |
| Dentist | € 115 | Visit | PECUNIA[1] |
| Optician/Optometrist | - | - | |
| **Diagnostic tests** | | | |
| Full lung function | - | - | |
| Chest x-ray | - | - | |
| Echocardiogram | - | - | |
| Electrocardiogram (ECG) | - | - | |
| Blood gas test | - | - | |
| Magnetic Resonance Image (MRI) | - | - | |
| CT/CAT Scan | - | - | |
| Blood test | - | - | |
| **Equipment** | | | |
| Ambulator oxygen (cylinders) | - | - | |
| Long term oxygen therapy (oxygen concentrator) | - | - | |
| Non-invasive ventilation (or CPAP) | - | - | |
| Walking aid (e.g., Rollator) | € 60 | Item | Bock et al.[22] |
| Wheelchair - manual | - | - | |
| Wheelchair - electric | - | - | |
| Feeding pump | - | - | |

| | | | |
|---|---|---|---|
| Commode | - | - | |
| Special bed | - | - | |
| Bathroom or toilet adapted | - | - | |

### 3.1.4 Italy

We were unable to identify a definitive unit cost database for Italy. We used results from EU HCSCD, and to address extant gaps we searched PubMed (MEDLINE) for recent economic evaluations. Local currency is EUR (€); we standardised costs to 2022 using the OECD Health CPI for Italy.[21]

*Table 4 Identified unit costs for Italy, 2022 €*

| | Unit cost (2022 €) | Per | Source |
|---|---|---|---|
| **Hospital** | | | |
| Hospital inpatient ward | € 414 | Night | Pöhlmann et al.[23] Trevisan et al.[24‡] |
| Intensive/Critical care ward | € 1,354 | Night | Mastrogianni et al.[14] |
| Specialist rehab ward/unit | - | - | - |
| **Other residential** | | | |
| Hospice | - | - | - |
| Nursing or residential home | - | - | - |
| Respite care setting | - | - | - |
| **Outpatient hospital** | | | |
| Emergency ambulance | € 215 | Visit | Espín et al.[2] |
| Emergency dept (A&E) | € 289 | Visit | Espín et al.[2] |
| Pulmonary rehab | - | - | - |
| Specialist (e.g., respiratory, cardiac, oncology) | - | - | - |
| Other outpatient visit | - | - | - |
| Day hospital (e.g., cardiac, oncology, respirator) | - | - | - |
| **Community-based** | | | |
| General Practitioner/ Primary Care Physician | € 64 | Hour | Espín et al.[2] |
| General practice nurse | € 13 | Hour | Espín et al.[2] |
| District/Public health/ Community nurse | € 13 | Hour | Espín et al.[2] |
| Physiotherapist | - | - | - |
| Occupational therapist | - | - | - |
| Speech & language therapist | - | - | - |
| Dietitian | € 13 | Visit | Pöhlmann et al.[23] |
| Psychologist | - | - | - |
| Psychiatrist | € 23 | Visit | Pöhlmann et al.[23] |
| Pulmonary rehab | - | - | - |

‡ Total cost of admission in Pöhlmann was €4,455 (2012 values). We calculated daily cost based on most recently available LOS data for 2018 (https://pubmed.ncbi.nlm.nih.gov/36941535/).

| | | | |
|---|---|---|---|
| Specialist palliative care doctor/consultant | € 22 | Hour | Espín et al.[2] |
| Palliative care nurse | - | - | - |
| Home palliative care/hospice | - | - | - |
| Social worker | - | - | - |
| Home help/care worker/ health care assistant | - | - | - |
| Dentist | - | - | - |
| Optician/Optometrist | € 23 | Visit | Pöhlmann et al.[23] |
| **Diagnostic tests** | | | |
| Full lung function | - | - | - |
| Chest x-ray | - | - | - |
| Echocardiogram | - | - | - |
| Electrocardiogram (ECG) | - | - | - |
| Blood gas test | - | - | - |
| Magnetic Resonance Image (MRI) | - | - | - |
| CT/CAT Scan | € 99 | Hour | Espín et al.[2] |
| Blood test | - | - | - |
| **Equipment** | | | |
| Ambulator oxygen (cylinders) | - | - | - |
| Long term oxygen therapy (oxygen concentrator) | - | - | - |
| Non-invasive ventilation (or CPAP) | - | - | - |
| Walking aid (e.g., Rollator) | - | - | - |
| Wheelchair - manual | - | - | - |
| Wheelchair - electric | - | - | - |
| Feeding pump | - | - | - |
| Commode | - | - | - |
| Special bed | - | - | - |
| Bathroom or toilet adapted | - | - | - |

### 3.1.5 Poland

We were unable to identify a definitive unit cost database for Poland. EU HCSCD estimated unit costs for Poland. We used results from EU HCSCD, and to address extant gaps we searched PubMed (MEDLINE) for recent economic evaluations. Local currency is PL (zł) but the only available costs were reported in EUR; we standardised costs to 2022 using the OECD Health CPI for Poland.[21]

*Table 5 Identified unit costs for Italy, 2022 €*

| | Unit cost (2022 €) | Per | Source |
|---|---|---|---|
| **Hospital** | | | |
| Hospital inpatient ward | €245 | Night | WHO CHOICE[3] |
| Intensive/Critical care ward | €451 | Night | Espín et al.[2] |
| Specialist rehab ward/unit | - | - | - |
| **Other residential** | | | |
| Hospice | - | - | - |

| | | | |
|---|---|---|---|
| Nursing or residential home | - | - | - |
| Respite care setting | - | - | - |
| **Outpatient hospital** | | | |
| Emergency ambulance | - | - | - |
| Emergency dept (A&E) | - | - | - |
| Pulmonary rehab | - | - | - |
| Specialist (e.g., respiratory, cardiac, oncology) | €26 | Visit | Espín et al.[2] |
| Other outpatient visit | €27 | Visit | WHO CHOICE[3] |
| Day hospital (e.g., cardiac, oncology, respirator) | - | - | - |
| **Community-based** | | | |
| General Practitioner/ Primary Care Physician | - | - | - |
| General practice nurse | - | - | - |
| District/Public health/ Community nurse | - | - | - |
| Physiotherapist | - | - | - |
| Occupational therapist | - | - | - |
| Speech & language therapist | - | - | - |
| Dietitian | - | - | - |
| Psychologist | - | - | - |
| Psychiatrist | - | - | - |
| Pulmonary rehab | - | - | - |
| Specialist palliative care doctor/consultant | - | - | - |
| Palliative care nurse | - | - | - |
| Home palliative care/hospice | - | - | - |
| Social worker | - | - | - |
| Home help/care worker/ health care assistant | - | - | - |
| Dentist | - | - | - |
| Optician/Optometrist | - | - | - |
| **Diagnostic tests** | | | |
| Full lung function | - | - | - |
| Chest x-ray | - | - | - |
| Echocardiogram | - | - | - |
| Electrocardiogram (ECG) | - | - | - |
| Blood gas test | - | - | - |
| Magnetic Resonance Image (MRI) | - | - | - |
| CT/CAT Scan | €100 | Test | Espín et al.[2] |
| Blood test | - | - | - |
| **Equipment** | | | |
| Ambulator oxygen (cylinders) | - | - | - |
| Long term oxygen therapy (oxygen concentrator) | - | - | - |
| Non-invasive ventilation (or CPAP) | - | - | - |
| Walking aid (e.g., Rollator) | - | - | - |
| Wheelchair - manual | - | - | - |
| Wheelchair - electric | - | - | - |
| Feeding pump | - | - | - |
| Commode | - | - | - |
| Special bed | - | - | - |

| Bathroom or toilet adapted | - | - | - |
|---|---|---|---|

### 3.1.6 Australia

We were unable to identify a definitive unit cost database for Australia, which operates a federal healthcare system. Based on discussions with study collaborators, with our professional networks, and published studies, we identified multiple relevant sources of unit costs for different parts of the health system. The two most important were the Independent Health and Aged Care Pricing Authority (IHACPA),[25] and Medicare Benefits Schedule.[26] Local currency is AUD (AU$); we standardised costs to 2022 using the national health CPI.[27]

*Table 6 Identified unit costs for Australia, 2022 AU$*

| | Unit cost (2022 AU$) | Per | Source |
|---|---|---|---|
| **Hospital** | | | |
| Hospital inpatient ward | $2,271 | Night | IHACPA[25] |
| Intensive/Critical care ward | $5,704 | Night | Hicks et al. (2019)[28] |
| Specialist rehab ward/unit | $1,284 | Night | |
| **Other residential** | | | |
| Hospice | - | - | |
| Nursing or residential home | $352 | Night | IHACPA[25] |
| Respite care setting | $373 | Night | IHACPA[25] |
| **Outpatient hospital** | | | |
| Emergency ambulance | - | - | |
| Emergency dept (A&E) | $789 | Visit | IHACPA[25] |
| Pulmonary rehab | $422 | Visit | IHACPA[25] |
| Specialist (e.g., respiratory… | $422 | Visit | IHACPA[25] |
| Other outpatient visit | $262 | Visit | IHACPA[25] |
| Day hospital (e.g., cardiac… | $422 | Visit | IHACPA[25] |
| **Community-based** | | | |
| General Practitioner/… | $80 | Visit | Medicare Benefits Schedule[26] |
| General practice nurse | $50 | Visit | Medicare Benefits Schedule[26] |
| District/Public health/ … | $50 | Visit | Medicare Benefits Schedule[26] |
| Physiotherapist | $238 | Hour | Farag et al. (2013)[29] |
| Occupational therapist | $97 | Visit | Farag et al. (2013)[29] |
| Speech & language therapist | - | - | |
| Dietitian | - | - | |
| Psychologist | $250 | Hour | Farag et al. (2013)[29] |
| Psychiatrist | $182 | Visit | Medicare Benefits Schedule[26] |
| Pulmonary rehab | - | - | |
| Specialist palliative care … | $189 | Hour | Medicare Benefits Schedule[26] |
| Palliative care nurse | - | - | |
| Home palliative care/hospice | - | - | |
| Social worker | - | - | |

| | | | |
|---|---|---|---|
| Home help/care worker/ … | $55 | Hour | Medicare Benefits Schedule[26] |
| Dentist | $76 | Visit | Medicare Benefits Schedule[26] |
| Optician/Optometrist | - | - | |
| **Diagnostic tests** | | | |
| Full lung function | - | - | |
| Chest x-ray | $111 | Test | Medicare Benefits Schedule[26] |
| Echocardiogram | $239 | Test | Medicare Benefits Schedule[26] |
| Electrocardiogram (ECG) | $239 | Test | Medicare Benefits Schedule[26] |
| Blood gas test | - | - | |
| Magnetic Resonance Image … | $593 | Test | Medicare Benefits Schedule[26] |
| CT/CAT Scan | $515 | Test | Medicare Benefits Schedule[26] |
| Blood test | - | - | |
| **Equipment** | | | |
| Ambulator oxygen (cylinders) | - | - | |
| Long term oxygen therapy … | - | - | |
| Non-invasive ventilation … | - | - | |
| Walking aid (e.g., Rollator) | - | - | |
| Wheelchair - manual | - | - | |
| Wheelchair - electric | - | - | |
| Feeding pump | $157 | Item | Medicare Benefits Schedule[26] |
| Commode | - | - | |
| Special bed | - | - | |
| Bathroom or toilet adapted | - | - | |

### 3.1.7 New Zealand

We were unable to identify a definitive unit cost database for New Zealand. The local currency is New Zealand dollar (NZD). In the context of constraints within our own study (the Australia and New Zealand populations were not included in the original research plan), we imputed all unit costs for New Zealand (see below).

## 3.2 Standardising units and currencies, and imputing missing unit costs for formal care

### 3.2.1 Overview

There were three challenges to finalising a unit cost database from the parameters identified in §3.1. First, identified unit costs sometimes varied in the relevant unit, e.g. €$x$ per GP hour in one country and €$y$ per visit in another. Second, identified unit costs in the UK and Australia were expressed in a different currency to those for other countries. Third, unit costs were missing for at least one service in all countries, and for all services in New Zealand.

To standardise units of service, we identified inconsistencies (highlighted orange in §3.1) and addressed on a case-by-case basis in the context of the literature. To standardise identified costs by currency, we adjusted UK and Australia costs to Euros using purchasing power parities (PPP). To estimate unit costs for imputation where a cost was not identified, we combined OECD relative price

data for the health care sector[30] with unit cost data that we identified as part of the study (§3.1 above).

### 3.2.2 Standardisation of units of service

In §3.1, reported unit costs for the most part corresponded to a consistent set of units of service:

- For hospital and other residential, per night;
- For outpatient hospital and community-based, per visit;
- For diagnostic tests, per test;
- For equipment, per treatment or item.

(NB some sources may have used different terminology, e.g. per contact instead of per visit, and we have standardised such terms in our tables where we interpret these to be the same, for ease of understanding).

However, in some cases costs were not reported per unit of service but instead per hour. These instances are marked above in orange and presented again below. Based on prior investigator experience[11,19] and published data,[26] we reshaped hourly rates on the following principles:

- GP clinic engagements with doctors and nurses last an estimated 15 minutes
- Community doctor visits last an estimated 30 minutes
- Community nurse visits last an estimated 45 minutes
- Homecare visits last an estimated 60 minutes
- Scans last an estimated 15 minutes
- Psychologist appointments last an estimated 60 minutes
- Allied and community health appointments last an estimated 30 minutes
- Total ambulance travelling time from receiving the call to picking up the patient to arriving at hospital is an estimated 30 minutes

Consequently, hourly unit costs were reweighted as follows:

*Table 7 List of services where unit of service was per hour*

| UK | Weight for hourly unit cost |
|---|---|
| General practice nurse | 0.25 |
| District/Public health/ Community nurse | 0.75 |
| Physiotherapist | 0.5 |
| Occupational therapist | 0.5 |
| Speech & language therapist | 1 |
| Dietitian | 0.5 |
| Psychologist | 1 |
| Palliative care nurse | 0.75 |
| Home palliative care/hospice | 0.5 |
| Social worker | 0.5 |
| Home help/care worker/ health care assistant | 1 |

| | |
|---|---|
| Dentist | 0.5 |
| Optician/Optometrist | 0.5 |
| **Germany** | |
| Emergency ambulance | 0.5 |
| **Italy** | |
| General Practitioner/ Primary Care Physician | 0.25 |
| General practice nurse | 0.75 |
| District/Public health/ Community nurse | 0.75 |
| Specialist palliative care doctor/consultant | 0.5 |
| CT/CAT Scan | 0.25 |
| **Australia** | |
| Physiotherapist | 0.5 |
| Specialist palliative care … | 0.5 |
| Home help/care worker/ … | 1 |

This approach has an important limitation that we are assuming constant time of engagement across countries, but we are unaware of comparable cross-country data that supports a more sophisticated approach.

### 3.2.3 Standardisation of identified costs to 2022 Euro

The OECD reported the following PPPs for GDP for one US dollar in 2022[31]:

*Table 8 Purchasing power parities for the currencies of participating countries in 2022, per the OECD*

| | **PPP** |
|---|---|
| Australia (ASD) | 1.372115 |
| Eurozone (EUR) | 0.685062 |
| United Kingdom (GBP) | 0.651256 |

Therefore, to express all identified costs in one currency for one year (2022€), we converted other currencies as follows:

- ASD: multiply by [0.685062/1.372115 = 0.4992],
- GBP: multiply by [0.685062/0.651256 = 1.052].

### 3.2.4 Imputation of missing unit costs

The most recently available OECD relative price data report the following for participating countries:[30]

*Table 9 Relative price levels in the health care sector in 2017, per the OECD*

| | **Relative price level (OECD average = 100)** |
|---|---|

| | |
|---|---|
| Australia | 115 |
| Germany | 79 |
| Ireland | 125 |
| Italy | 95 |
| New Zealand | 84 |
| Poland | 34 |
| United Kingdom | 96 |

For each category, we combined *identified* unit costs with OECD relative prices to calculate country-specific *identified* unit costs at the OECD average price level. To impute missing unit costs, we took the median of all *identified* unit costs at the average OECD price level and revised this median value for the local country price. Consider this worked example:

### *Worked example: nursing home/residential care*

We identified in §3.1 the cost of a night in a nursing or residential care home for the UK, Ireland, Germany and Australia in their local currencies; we are missing a unit cost for this service for Italy and Poland.

Identified unit costs are entered into Row 1 of the table below. We adjusted local currency values by the PPP exchange rate (Row 2) to estimate identified unit costs in Euros for 2022 (Row 3). We adjusted those unit costs according to relative health care prices (Row 4) and took the median of the four values, €167 (Row 5). Finally, for Italy and Poland we took the median value from Row 5 and adjusted by OECD relative prices (Row 4) to estimate the unit cost (Row 6).

*Table 10 Worked example of estimating unit costs for imputation: night in a nursing home or residential care*

| | | UK | Ire | Ger | Ita | Pol | Aus | Median |
|---|---|---|---|---|---|---|---|---|
| 1 | Local currency | £165 | € 148 | € 146 | - | - | $352 | |
| 2 | PPP EXR | 1.052 | | | | | 0.4992 | |
| 3 | In 2022€ | € 174 | € 148 | € 146 | - | - | € 176 | |
| 4 | Health care prices | 96 | 125 | 79 | 95 | 34 | 115 | |
| 5 | OECD average | € 181 | € 118 | € 185 | - | - | € 153 | € 167 |
| 6 | UC in analysis | € 174 | € 148 | € 146 | € 158 | € 57 | € 176 | |

In summary:

- Where a unit cost was identified in Euros for a specific service in Ireland, Germany, Italy or Poland, this cost was used for the relevant service and country. Marked green in the example above.

- Where a unit cost was identified for a specific service in the UK or Australia, this cost was converted to Euros using PPP exchange rates and used for the relevant service and country. Marked orange in the table above.

- Where a unit cost was not identified for a specific service in any country, we adjusted all identified unit costs for that service using relative health care prices, identified the median value at the OECD average, and adjusted that median value to relative health care prices in the relevant country. Marked purple in the table above.

- For New Zealand, we did not identify unit costs in §3.1. Therefore, all unit costs for New Zealand were calculated using the approach marked purple in the table above.

*Justification*

This approach prioritises first unit costs where these have been identified in a relevant country, and then estimates for imputation those values that represent our best estimate of what a given service cost in a given country, taking into account the variation in health care prices across participating countries.[32] The most significant weakness of this approach is that both identified and imputed unit cost values are associated with an unmeasured amount of uncertainty. In principle, this might be tackled using multiple imputation,[33] but such imputation requires either multiple unit costs and/or a plausible distribution for unit costs in each category of service in each participating country. We were not able to identify sufficient suitable data for this exercise.

Additionally, we note a number of contextual factors worth bearing in mind when interpreting these costs. First, COVID-19 and the associated delays made it much more difficult to engage with potential sources, and many parts of health systems diverted staff away from routine and admin roles to managing the changed service needs. Second, in the end recruitment to the trial varied hugely between countries, and the priority became to put resources into developing the best (feasible) cost vectors for those countries. Third, a benefit of this study has been to reveal more clearly the patterns of available and unavailable unit costs data across the different countries, and showing that there are important gaps that need to be filled outside of the limited resources available in a clinical trial. Fourth, once the patterns of service use were analysed it became clear that variation is strongly driven by inpatient care and the associated investigations and treatments (plus a few nonhospital services). The team therefore concentrated on ensuring that the best feasible estimates were available for these services.

## 3.3 Unit cost database in primary analysis

Following the procedure outlined above, the unit costs employed in primary analysis are provided below, imputed costs marked in grey/orange:

*Table 11 Unit cost database for primary analysis, 2022 €*

| | UK | Ireland | Germany | Italy | Poland | Aus | NZ |
|---|---|---|---|---|---|---|---|
| **Hospital** | | | | | | | |
| Hospital inpatient | €654 | €2,024 | €648 | €414 | €245 | €1,134 | €647 |
| Intensive/Critical care | €2,094 | €3,151 | €1,506 | €1,354 | €451 | €2,848 | €1,717 |
| Specialist rehab | €616 | €750 | €474 | €570 | €204 | €641 | €504 |
| **Other residential** | | | | | | | |
| Hospice | €503 | €786 | €455 | €547 | €196 | €663 | €484 |
| Nursing or residential | €174 | €148 | €146 | €158 | €57 | €176 | €140 |
| Respite care setting | €105 | €170 | €107 | €129 | €46 | €186 | €114 |
| **Outpatient hospital** | | | | | | | |
| Emergency ambulance | €286 | €373 | €315 | €215 | €101 | €343 | €250 |
| Emergency dept (A&E) | €124 | €321 | €135 | €289 | €87 | €394 | €216 |
| Pulmonary rehab | €205 | €190 | €145 | €174 | €62 | €211 | €154 |
| Specialist | €205 | €184 | €74 | €140 | €26 | € 211 | €124 |
| Other outpatient visit | € 146 | € 184 | €116 | €140 | €50 | € 131 | €124 |
| Day hospital | € 817 | € 841 | €421 | €573 | €205 | € 211 | €506 |
| **Community-based** | | | | | | | |
| GP | €43 | €43 | €23 | €16 | €12 | €40 | €29 |
| General practice nurse | €10 | €17 | €16 | €10 | €5 | €25 | €11 |
| District nurse | €31 | €44 | €21 | €10 | €9 | €25 | €23 |
| Physiotherapist | €26 | €34 | €18 | €26 | €9 | €59 | €23 |
| Occupational therapist | €26 | €34 | €42 | €33 | €12 | €48 | €29 |
| Speech & language | €53 | €68 | €43 | €52 | €19 | €63 | €46 |
| Dietitian | €26 | €34 | €34 | €26 | €9 | €32 | €23 |
| Psychologist | €53 | €104 | €66 | €79 | €28 | €125 | €70 |
| Psychiatrist | €76 | €231 | €50 | €75 | €27 | €91 | €66 |
| Pulmonary rehab | €61 | €58 | €43 | €52 | €19 | €63 | €46 |
| SPC doctor/consultant | €39 | €121 | €32 | €11 | €14 | €47 | €34 |
| Palliative care nurse | €93 | €45 | €53 | €63 | €23 | €76 | €56 |
| Home palliative care | €62 | €111 | €61 | €73 | €26 | €88 | €64 |
| Social worker | €24 | €30 | €19 | €23 | €8 | €28 | €21 |
| Home help | €33 | €35 | €22 | €27 | €10 | €27 | €24 |
| Dentist | €57 | €95 | €115 | €64 | €23 | €38 | €57 |
| Optician/Optometrist | €53 | €68 | €43 | €52 | €19 | €63 | €46 |
| **Diagnostic tests** | | | | | | | |
| Full lung function | €14 | €18 | €11 | €14 | €5 | €16 | €12 |
| Chest x-ray | €60 | €69 | €44 | €53 | €19 | €55 | €46 |
| Echocardiogram | €219 | € 207 | € 131 | € 158 | €56 | €119 | € 139 |
| Electrocardiogram | €34 | €87 | €55 | €66 | €24 | €119 | €58 |
| Blood gas test | €32 | €41 | €26 | €31 | €11 | €38 | €28 |
| MRI | €488 | € 479 | € 303 | € 364 | € 130 | €296 | € 322 |

| | | | | | | | |
|---|---|---|---|---|---|---|---|
| CT/CAT Scan | €168 | € 249 | € 158 | €25 | €100 | € 257 | € 168 |
| Blood test | €32 | €41 | €26 | €31 | €11 | €38 | €28 |
| **Equipment** | | | | | | | |
| Ambulator oxygen | €96 | € 125 | €79 | €95 | €34 | € 115 | €84 |
| Long term oxygen | €113 | € 147 | €93 | € 111 | €40 | € 135 | €98 |
| Non-invasive vent. | €458 | € 596 | € 377 | € 453 | € 162 | € 548 | € 400 |
| Walking aid | €60 | €86 | €59 | €65 | €23 | €79 | €58 |
| Wheelchair - manual | €112 | € 145 | €92 | € 110 | €39 | € 134 | €98 |
| Wheelchair - electric | €523 | € 681 | € 430 | € 517 | € 185 | € 626 | € 457 |
| Feeding pump | €65 | €85 | €54 | €65 | €23 | €78 | €57 |
| Commode | €26 | €34 | €22 | €26 | €9 | €32 | €23 |
| Special bed | €2,880 | €3,750 | € 2,370 | € 2,850 | € 1,020 | € 3,450 | € 2,520 |
| Bathroom or toilet | €627 | € 816 | € 516 | € 620 | € 222 | € 751 | € 549 |

Some service names shortened. See also §3.1.

*Miscellaneous formal costs*

For 'other healthcare professionals in the community', we categorised manually the recorded text responses into one of three groups: physician provider, nurse provider or other. Respectively we applied the unit costs of outpatient hospital appointment, public health nurse and the median unit cost of allied health professionals. For 'other equipment' we categorised manually the recorded text responses into one of five groups: basic (e.g. stool), low-tech (e.g. installed guard rails), moderate (e.g. nebuliser), substantial not-high-tech item (e.g. reclining chair, exercise equipment) and high-tech (e.g. electric mobility scooter). Respectively we applied the unit costs of commode, walking aid, long-term oxygen, wheelchair, special bed.

## 3.4 Identified unit costs for **informal** care in participating countries

In our CSRI (Appendix 2), we collected data on six types of unpaid informal care:

i. Personal care
ii. Help with medications
iii. Help inside the home
iv. Help outside the home
v. On-call
vi. Other types of help

The challenges specifying a unit cost have been extensively covered in the literature, and four main approaches identified: the opportunity cost approach, the replacement cost approach, contingent valuation, and conjoint analysis.[4] Since contingent valuation and conjoint analysis require primary data collection (willingness to pay and preference elicitation respectively), and we were not able to

identify sources of informal care unit costs using these methods for all participating countries, we restricted our analyses to the opportunity cost approach, and the replacement cost approach.

An additional challenge is how to cost different types of informal care. The replacement cost approach is more obviously relevant to *active care* such as (i-iv) above; for example, healthcare systems would plausibly provide a home help package for personal care in lieu of an available family carer. It is less plausible that they would send someone simply "to spend time" with a patient, but that time nevertheless has an opportunity cost to the carer. In primary analysis we categorised (i-iv) above as *active care* and costed using a replacement cost approach, defined as the cost of a home care package to cover reported hours, and we categorised (v-vi) as *other types of support* and costed using opportunity costs, defined as the median hourly wage in each country in 2022. In the context of potential over-reporting of unpaid care,[34] in particular one person caring for more than 24 hours per day, we capped *active care* at 16 hours per day and [*active care* + *other types of support*] at 24 hours per day.

Hourly replacement costs, defined as the cost of home help to cover reported hours, were identified as follows from §3.3:

*Table 12 Identified unit costs for informal care, replacement cost approach, 2022 €*

| | UK | Ireland | Germany | Italy | Poland | Aus | NZ |
|---|---|---|---|---|---|---|---|
| Replacement costs | €33 | €35 | €22 | €27 | €10 | €27 | €24 |

We defined hourly opportunity costs as the median hourly wage in each country in 2022. We sourced real average annual wages in 2022 USD[35] and average hours worked annually[36] from the OECD. We combined these data to get average hourly wage in USD and multiplied by 0.685062[31] to express in Euro.

*Table 13 Identified unit costs for informal care, opportunity cost approach, 2022 €*

| | UK | Ireland | Germany | Italy | Poland | Aus | NZ |
|---|---|---|---|---|---|---|---|
| Average wage (USD) | $53,985 | $52,243 | $58,940 | $44,893 | $36,897 | $59,408 | $50,722 |
| Average hours worked | 1,532 | 1,657 | 1,341 | 1,694 | 1,815 | 1,707 | 1,748 |
| Hourly wage (2022€) | €24 | €22 | €30 | €18 | €14 | €24 | €20 |

*Hourly wage (2022€) = (Average wage USD/hours worked)* 0.685062

## 3.5 References

1. Mayer S, Berger M, Konnopka A, et al. In Search for Comparability: The PECUNIA Reference Unit Costs for Health and Social Care Services in Europe. *Int J Environ Res Public Health*. Mar 16 2022;19(6)doi:10.3390/ijerph19063500

2. Espín J, Špacírová Z, Rovira J, Epstein D, Olry de Labry Lima A, García-Mochón L. Development of the European Healthcare and Social Cost Database (EU HCSCD) for use in economic evaluation of healthcare programs. *BMC Health Services Research*. 2022/03/27 2022;22(1):405. doi:10.1186/s12913-022-07791-z

3. Stenberg K, Lauer JA, Gkountouras G, Fitzpatrick C, Stanciole A. Econometric estimation of WHO-CHOICE country-specific costs for inpatient and outpatient health service delivery. *Cost Eff Resour Alloc*. 2018;16:11. doi:10.1186/s12962-018-0095-x

4. Hu B, Cartagena-Farias J, Brimblecombe N, Jadoolal S, Wittenberg R. Projected costs of informal care for older people in England. *The European Journal of Health Economics*. 12/12 2023:1-14. doi:10.1007/s10198-023-01643-1

5. Personal Social Services Research Unit. Unit Costs of Health and Social Care programme. University of Kent. https://www.pssru.ac.uk/unitcostsreport/

6. National Health Service (England). Data from: National Cost Collection for the NHS. 2024.

7. Office for National Statistics. Data from: CPI WEIGHTS 06 : HEALTH. 2023.

8. Round J, Jones L, Morris S. Estimating the cost of caring for people with cancer at the end of life: A modelling study. *Palliat Med*. Dec 2015;29(10):899-907. doi:10.1177/0269216315595203

9. Hassiotis A, Poppe M, Strydom A. Appendix 4, Unit costs for health, social care and criminal justice. *Positive behaviour support training for staff for treating challenging behaviour in people with intellectual disabilities: a cluster RCT*. NIHR Journals Library; 2018.

10. National Institute for Health and Care Research. Data from: Online SoECAT Guidance. 2024.

11. Smith S, Jiang J, Normand C, O'Neill C. Unit costs for non-acute care in Ireland 2016-2019. *HRB Open Res*. 2021;4:39. doi:10.12688/hrbopenres.13256.1

12. Central Statistics Office. Data from: Consumer Price Index. 2024.

13. May P, Moriarty F, Hurley E, et al. Formal health care costs among older people in Ireland: methods and estimates using The Irish Longitudinal Study on Ageing (TILDA) [version 1; peer review: 1 approved with reservations]. *HRB Open Research*. 2023;6(16)doi:10.12688/hrbopenres.13692.1

14. Mastrogianni M, Galanis P, Kaitelidou D, Konstantinou E, Fildissis G, Katsoulas T. Factors affecting adult intensive care units costs by using the bottom-up and top-down costing methodology in OECD countries: A systematic review. *Intensive Crit Care Nurs*. Oct 2021;66:103080. doi:10.1016/j.iccn.2021.103080

15. Keegan C, Brick A, Bergin A, Wren M-A, Henry E, Whyte R. Projections of expenditure for public hospitals in Ireland, 2018–2035, based on the Hippocrates Model. ESRI; 2020.

16. Carty P, O'Neill M, Teljeur C, Harrington P, Smith S, Ryan M. COPD Budget impact analysis - Management of Chronic Obstructive Pulmonary Disease in adults. Health Information and Quality Authority (HIQA) ; HRB-CICER; 2021.

17. Healthcare Pricing Office. *ABF 2022 Admitted Patient Price List*. 2023. https://www.hpo.ie/abf/ABF2022AdmittedPatientPriceList.pdf

18. Walsh B, Keegan C, Brick A, et al. Projections of expenditure for primary, community and long-term care in Ireland, 2019-2035, based on the Hippocrates model The Economic and Social Research Institute and the Minister for Health; 2021.

19. Brick A, Normand C, O'Hara S, et al. Economic Evaluation of Palliative Care in Ireland. Trinity College Dublin; 2015.

20. Smith S, Jiang JJ, Normand C, O'Neill C. The price of private dental services: results from a national representative survey of Ireland. *Irish Journal of Medical Science (1971 -)*. 2023/06/01 2023;192(3):973-983. doi:10.1007/s11845-022-03041-7

21. Organisation for Economic Development and Co-operation. Data from: Consumer price indices (CPIs) - Complete database. 2023.

22. Bock JO, Brettschneider C, Seidl H, et al. [Calculation of standardised unit costs from a societal perspective for health economic evaluation]. *Gesundheitswesen*. Jan 2015;77(1):53-61. Ermittlung standardisierter Bewertungssätze aus gesellschaftlicher Perspektive für die gesundheitsökonomische Evaluation. doi:10.1055/s-0034-1374621
23. Pöhlmann J, Norrbacka K, Boye KS, Valentine WJ, Sapin H. Costs and where to find them: identifying unit costs for health economic evaluations of diabetes in France, Germany and Italy. *Eur J Health Econ*. Nov 2020;21(8):1179-1196. doi:10.1007/s10198-020-01229-1
24. Trevisan C, Noale M, Zatti G, Vetrano DL, Maggi S, Sergi G. Hospital length of stay and 30-day readmissions in older people: their association in a 20-year cohort study in Italy. *BMC Geriatr*. Mar 21 2023;23(1):154. doi:10.1186/s12877-023-03884-4
25. Independent Health and Aged Care Pricing Authority. Costing. https://www.ihacpa.gov.au/costing
26. Australian Government. Medicare Benefits Schedule. https://www.mbsonline.gov.au/
27. Australian Bureau of Statistics. Price indexes and inflation. https://www.abs.gov.au/statistics/economy/price-indexes-and-inflation
28. Hicks P, Huckson S, Fenney E, Leggett I, Pilcher D, Litton E. The financial cost of intensive care in Australia: a multicentre registry study. *Med J Aust*. Oct 2019;211(7):324-325. doi:10.5694/mja2.50309
29. Farag I, Sherrington C, Ferreira M, Howard K. A systematic review of the unit costs of allied health and community services used by older people in Australia. *BMC Health Services Research*. 2013/02/20 2013;13(1):69. doi:10.1186/1472-6963-13-69
30. Organisation for Economic Development and Co-operation. *Health Care Prices*. 2020. https://www.oecd.org/health/health-systems/Health-Care-Prices-Brief-May-2020.pdf
31. Organisation for Economic Development and Co-operation. Data from: PPPs and exchange rates. 2023.
32. Manca A, Rice N, Sculpher MJ, Briggs AH. Assessing generalisability by location in trial-based cost-effectiveness analysis: the use of multilevel models. *Health Econ*. May 2005;14(5):471-85. doi:10.1002/hec.914
33. Grieve R, Cairns J, Thompson SG. Improving costing methods in multicentre economic evaluation: the use of multiple imputation for unit costs. *Health Econ*. Aug 2010;19(8):939-54. doi:10.1002/hec.1531
34. Elayan S, Angelini V, Buskens E, de Boer A. The Economic Costs of Informal Care: Estimates from a National Cross-Sectional Survey in The Netherlands. *The European Journal of Health Economics*. 2024/01/31 2024;doi:10.1007/s10198-023-01666-8
35. Organisation for Economic Development and Co-operation. Data from: Real average annual wages. 2024. *Paris*.
36. Organisation for Economic Development and Co-operation. Data from: Hours worked (indicator). 2024. doi:10.1787/47be1c78-en
