# Supplementary material for: The cost of drug repurposing: parallel economic evaluation of mirtazapine for severe breathlessness in the multinational BETTER-B trial
Source: BMC Health Serv Res. 2025 Nov 4;25:1442. doi: 10.1186/s12913-025-13605-9 (PMC12584416; doi:10.1186/s12913-025-13605-9)
Supplement: Supplementary file 4 — Supplementary Material 4 [file 12913_2025_13605_MOESM4_ESM.pdf]

Appendix 4: ICERs and other output for primary and sensitivity analysis

Overview

In primary analysis we evaluated the effect of mirtazapine on total (formal + informal costs) and QALYs to day 56, the primary trial endpoint. The statistical analysis plan pre-specified three sensitivity analyses to test key choices in our primary analysis:

- Using formal costs (instead of total (formal + informal) costs) to day 56 in analysis. This analysis tests the robustness of our key conclusions to choice of *perspective*.
- Using total costs and QALYs to day 180 (trial exit, instead of day 56) in analysis. This analysis tests the robustness of our key conclusions to choice of *timeframe*.
- Using one set of unit costs (UK costs), adjusted to relative prices in other countries, instead of the multiply-sourced international costs used in primary analysis (see Appendix 3). This analysis tests the robustness of our key conclusions to potential inconsistencies in unit cost calculation (e.g. if different sources take different approaches to costing employee benefits, overheads, etc).

Additionally we added a fourth sensitivity analysis based on recent methodological guidance:[1]

- We evaluated effects using seemingly unrelated regressions (distinct from unrelated regressions in primary analysis).

For each of the four analyses we repeated the missing data and statistical procedures reported in primary analysis, and we calculated incremental cost-effectiveness ratios (ICERs) and cost-effectiveness acceptability curves.

In this appendix we present the graphical output of the main analyses: ICERs, where that output parameter was defined, or otherwise the scatterplot of bootstrapped output.

Figure 1 Primary analysis

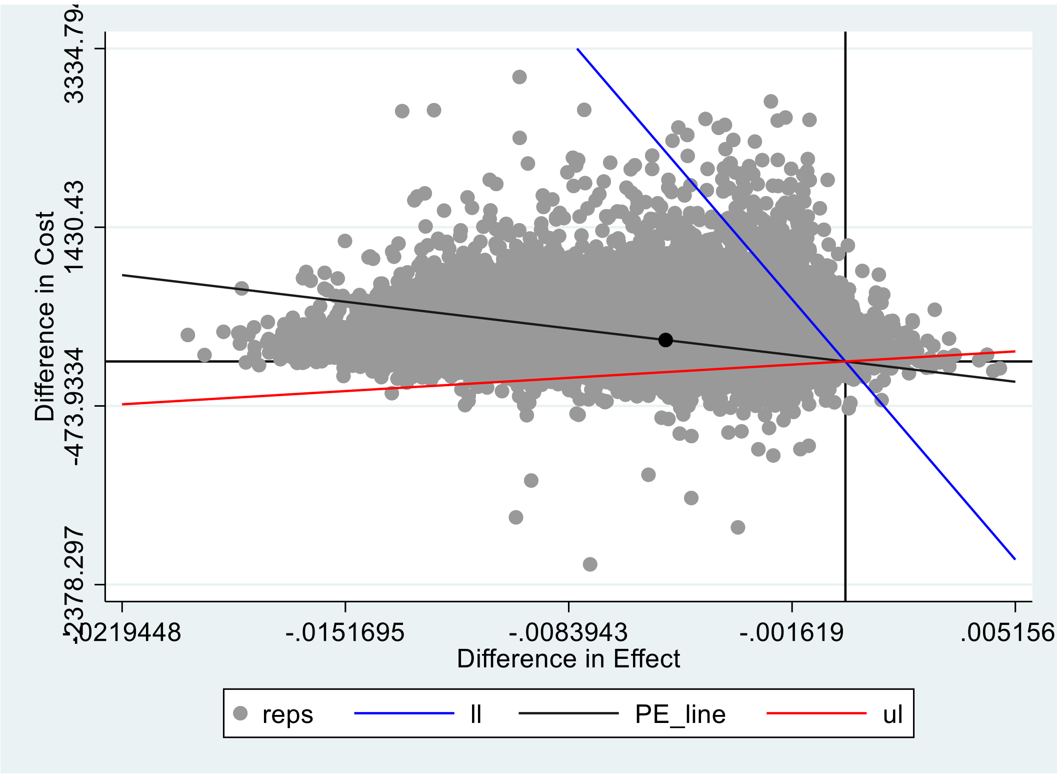

Figure 2 Sensitivity analysis (1): Perspective

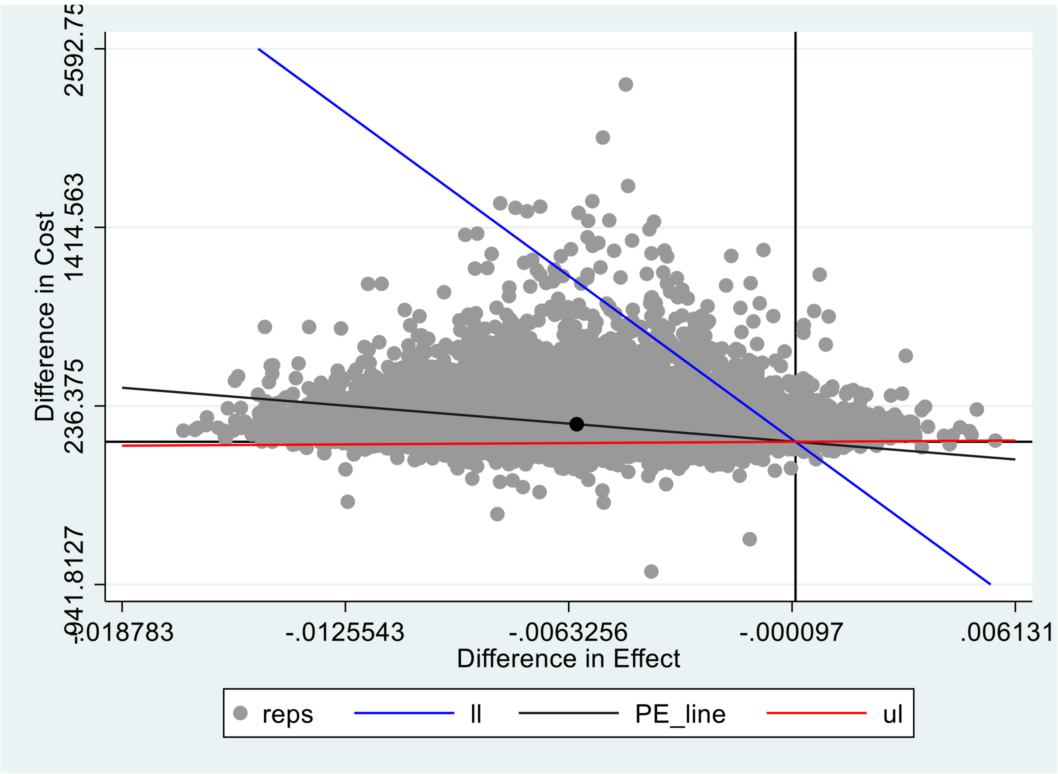

Figure 3 Sensitivity analysis (2): Timeframe (ICER not defined)

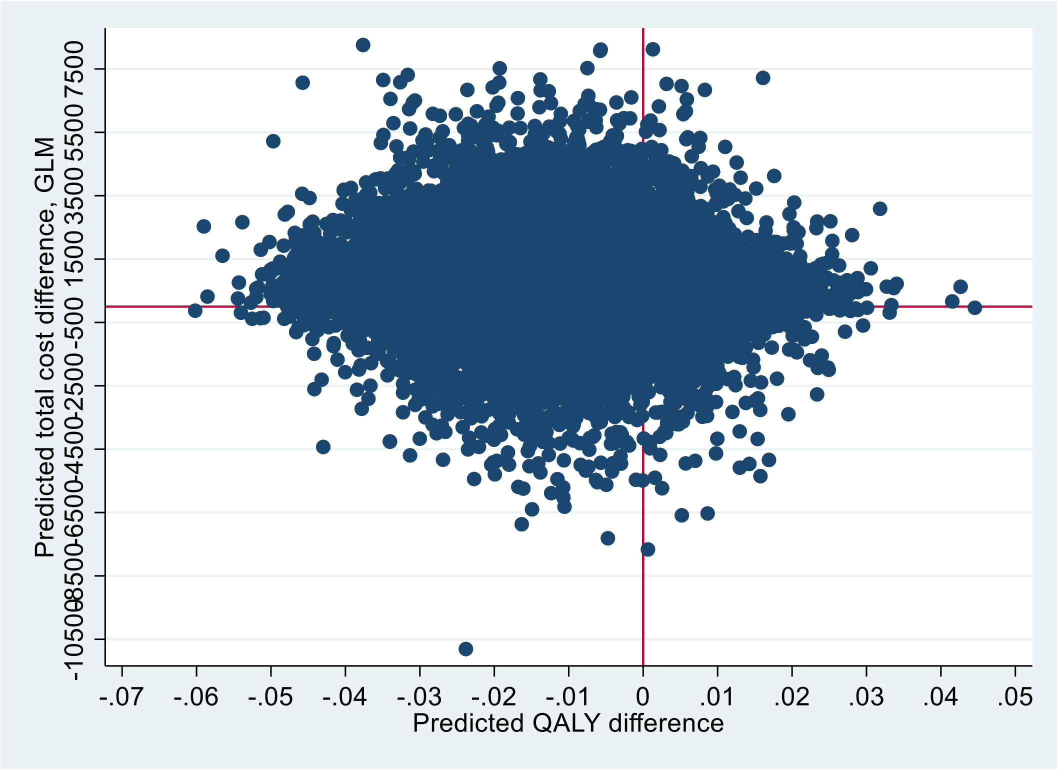

Figure 4 Sensitivity analysis (3): Unit cost inputs

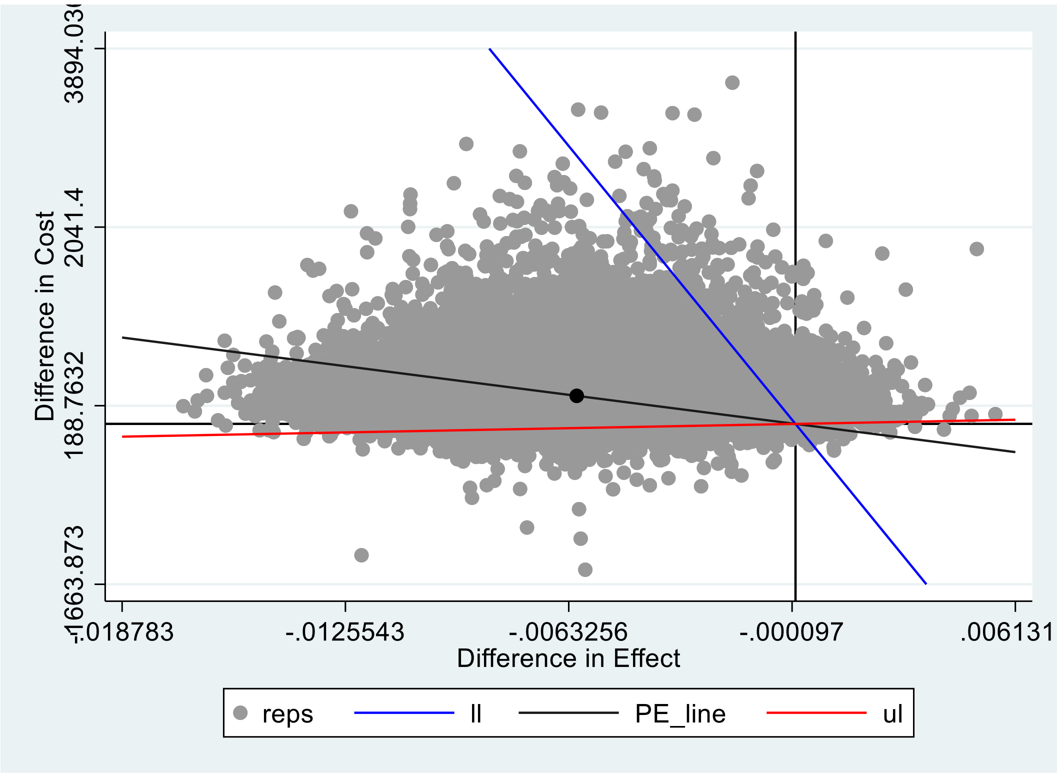

Figure 5 Sensitivity analysis (4): Modelling approach (ICER not defined)

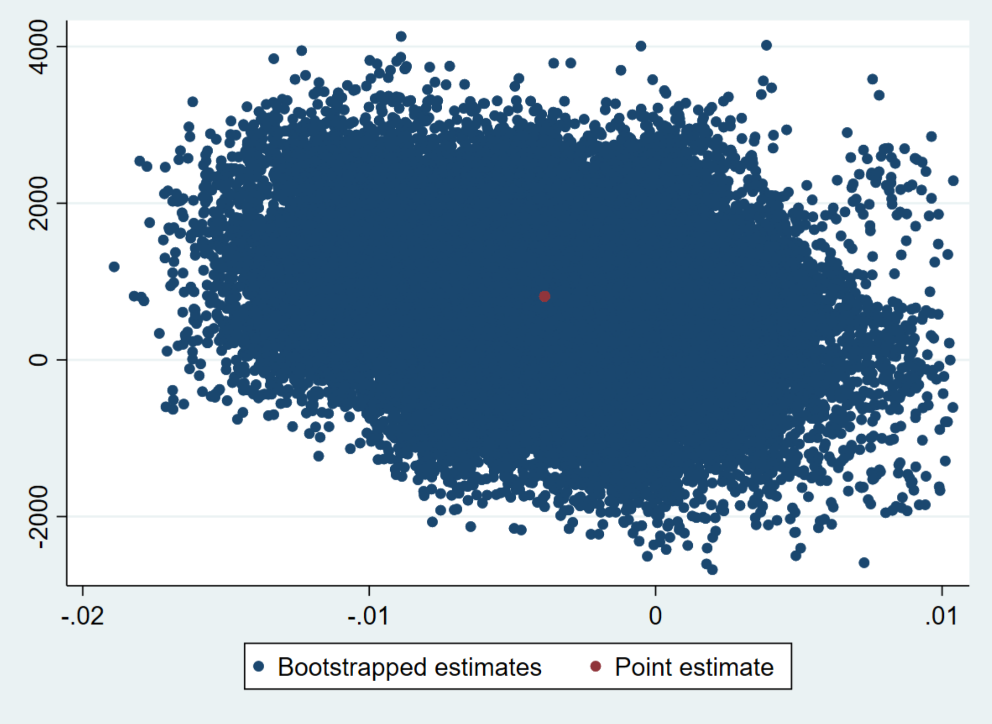

References

1. Mutubuki EN, El Alili M, Bosmans JE, et al. The statistical approach in trial-based economic evaluations matters: get your statistics together! *BMC Health Services Research*. 2021/05/19 2021;21(1):475. doi:10.1186/s12913-021-06513-1
