# Supplementary material for: The cost of drug repurposing: parallel economic evaluation of mirtazapine for severe breathlessness in the multinational BETTER-B trial
Source: BMC Health Serv Res. 2025 Nov 4;25:1442. doi: 10.1186/s12913-025-13605-9 (PMC12584416; doi:10.1186/s12913-025-13605-9)
Supplement: Supplementary file 5 — Supplementary Material 5 [file 12913_2025_13605_MOESM5_ESM.pdf]

Appendix 5: Parameters by country

Overview

The purpose of this appendix is to present some key model inputs by country. In §5.1, we present formal costs by country at primary and secondary endpoints, prior to multiple imputation. In §5.2, we present equivalent data for informal costs. In §5.3, we present equivalent data for health-related quality of life (HRQoL). All data presented are prior to multiple imputation.

5.1 Summary formal costs by country

5.1.1 Primary endpoint (day 56)

racountry	Summary of Total formal care costs Mean	Std. dev.	Freq.
UK	2593.1031	3024.0511	77
Ireland	3369.1379	5916.3049	17
Italy	712.21229	909.58062	34
Germany	3481.9833	6214.9301	24
Poland	351.52545	460.3216	13
Australia	11023.971	11217.788	5
New Zeala	2520.4585	2471.8642	8
Total	2497.643	4310.0398	178

5.1.2 Secondary endpoint (day 180)

racountry	Summary of Total formal care costs Mean	Std. dev.	Freq.
UK	7037.4682	9867.0331	62
Ireland	14008.154	15803.033	11
Italy	3741.2292	12849.543	30
Germany	12378.936	18119.415	24
Poland	1376.9472	2050.2564	8
Australia	23824.524	21937.43	5
New Zeala	5059.9206	4951.6645	8
Total	7907.8488	13307.31	148

5.2 Summary informal costs by country

5.2.1 Primary endpoint (day 56)

racountry	Summary of Total informal care costs Mean	Std. dev.	Freq.
UK	2543.2987	4632.7804	77
Ireland	587.11765	898.82422	17
Italy	494.05882	861.58888	34
Germany	684	1340.4602	24
Poland	1485.5385	2697.1234	13
Australia	8184	7995.9157	5
New Zeala	342	810.51095	8
Total	1696.6124	3707.6322	178

5.2.2 Secondary endpoint (day 180)

racountry	Summary of Total informal care costs Mean	Std. dev.	Freq.
UK	4270.5309	8370.4995	62
Ireland	1189.2722	1641.2081	11
Italy	1014.4425	2482.4917	30
Germany	1255.5262	2559.3122	24
Poland	4069.277	6525.2354	8
Australia	12800.813	11335.146	5
New Zeala	675.25397	1477.7261	8
Total	2975.5482	6555.6748	148

5.3 Summary HRQoL by country

5.3.1 Primary endpoint (day 56, max value=56/365)

	Percentiles	Smallest		
1%	.0179192	.0093031		
5%	.0425079	.0179192		
10%	.0553825	.0368615	Obs	173
25%	.0804221	.0392882	Sum of wgt.	173
50%	.1004627		Mean	.0989908
		Largest	Std. dev.	.0308221
75%	.1231157	.1514664		
90%	.1390609	.1533196	Variance	.00095
95%	.1466015	.1533196	Skewness	-.3138884
99%	.1533196	.1533196	Kurtosis	2.612564

racountry	Mean	Std. dev.	Freq.
UK	.08744516	.02778494	76
Ireland	.10092491	.03300899	17
Italy	.11948336	.02957333	33
Germany	.10953295	.02501557	22
Poland	.10696109	.0200158	13
Australia	.07850733	.0296938	5
New Zeala	.0897358	.03782206	7
Total	.09899083	.03082209	173

5.3.2 Secondary endpoint (day 180, max value= 180/365)

	Percentiles	Smallest		
1%	.0861614	.0766324		
5%	.1284052	.0861614		
10%	.1832666	.0936886	Obs	144
25%	.2433265	.1143821	Sum of wgt.	144
50%	.3315458		Mean	.3188834
		Largest	Std. dev.	.0998285
75%	.397922	.4843258		
90%	.443614	.487102	Variance	.0099657
95%	.4628501	.4928131	Skewness	-.4533926
99%	.4928131	.4928131	Kurtosis	2.494028

racountry	Mean	Std. dev.	Freq.
UK	.27927446	.09055298	60
Ireland	.30558845	.10723967	11
Italy	.39088777	.07370993	30
Germany	.34523403	.0872331	22
Poland	.36379527	.06452691	9
Australia	.25433101	.11958924	5
New Zeala	.27623899	.12898064	7
Total	.31888339	.0998285	144

References

1. Mutubuki EN, El Alili M, Bosmans JE, et al. The statistical approach in trial-based economic evaluations matters: get your statistics together! *BMC Health Services Research*. 2021/05/19 2021;21(1):475. doi:10.1186/s12913-021-06513-1
