# Supplementary material for: The cost of drug repurposing: parallel economic evaluation of mirtazapine for severe breathlessness in the multinational BETTER-B trial
Source: BMC Health Serv Res. 2025 Nov 4;25:1442. doi: 10.1186/s12913-025-13605-9 (PMC12584416; doi:10.1186/s12913-025-13605-9)
Supplement: Supplementary file 6 — Supplementary Material 6 [file 12913_2025_13605_MOESM6_ESM.pdf]

Appendix VI

Consortium members

Key role	Title	First name	Surname	One degree	Organisation, country
Health Economics	Dr	Peter	May	PhD	KCL, UK
Health Economics	Professor	Charles	Normand	DPhil	KCL, UK
Health Economist	Dr	Samantha	Smith	PhD	TCD, Ireland
Chief Investigator	Professor	Irene J	Higginson	FFPHM	KCL, UK
Project Manager	Dr	Adejoke O	Oluyase	PhD	KCL, UK
Co-Investigators/grant holders	Professor	Matthew	Maddocks	PhD	KCL, UK
Co-Investigators/grant holders	Dr	Massimo	Costantini	MD	KCL, UK
Co-Investigators/grant holders	Dr	Sabrina	Bajwah	PhD	KCL, UK
Clinical Trials Research Unit (Statistics)	Ms	Sarah T	Brown	MSc	Leeds, UK
Site Principal Investigator	Professor	Kathrin	Kahnert	PD Dr	University Hospital Muenchen, Germany
Co-Investigators/grant holders	Professor	Steffen T	Simon	PhD	KOELN, Germany
Co-Investigators/grant holders	Professor	Karen	Ryan	MD	UCD, Ireland
Co-Investigators/grant holders	Professor	David C	Currow	PhD	Wollongong, Australia
Site Principal Investigator	Professor	Miriam J	Johnson	MD	Hull, UK
Site Principal Investigator	Dr	Simon P	Hart	PhD	Hull, UK
Clinical Trials Research Unit (Statistics)	Ms	Hannah	Mather	MSc	Leeds, UK
Co-Investigators/grant holders	Professor	Małgorzata	Krajnik	PhD	Department of Palliative Care, Collegium Medicum in Bydgoszcz, Nicolaus Copernicus University in Toruń, Bydgoszcz, Poland
Site Principal Investigator	Dr	Silvia	Tanzi	PhD	Azienda AUSL-IRCCS Reggio Emilia, Italy

Qualitative Researcher	Dr	Luca	Ghirotto	PhD	Azienda AUSL-IRCCS Reggio Emilia, Italy
Other Clinicians	Professor	Charlotte E	Bolton	MD	Nottingham, UK
Site Principal Investigator	Dr	Piotr	Janowiak	PhD	Division of Pulmonology, Medical University of Gdańsk, Gdańsk, Poland
Site Project Manager	Dr	Elena	Turola	PhD	Azienda AUSL-IRCCS Reggio Emilia, Italy
Other Clinicians	Dr	Caroline J	Jolley	PhD	KCL, UK
Clinical Trials Research Unit	Mrs	Geraldine	Murden	MSc	Leeds, UK
Chair of the Ethics Advisory Board	Professor	Bobbie	Farsides	PhD	Sussex, UK
Co-Investigators/grant holders	Professor	Julia M	Brown	MSc	Leeds, UK

Other Better B Research Consortium Members

Co-Investigators/grant holders	Professor	Claudia	Bausewein	PhD	UMUEN, Germany
Site Principal Investigator	Dr	Perluigi	Donatelli	MD	Azienda AUSL-IRCCS Reggio Emilia, Italy
Site Investigator	Dr	Michael	Epton	PhD	Christchurch Hospital, New Zealand
Co-Investigators/grant holders	Dr	Rossella	Cianci	PhD	Universitia' Cattolica, Rome, Italy
Co-Investigators/grant holders	Professor	Ewa	Jassem	PhD	Division of Pulmonology, Medical University of Gdańsk, Gdańsk, Poland
Site Principal Investigator	Dr	Emer	Kelly	Associate Clinical Professor	SVUH, Ireland
Site Principal Investigator	Professor	Winfried	Randerath	MD	Bethanien Hospital, Germany
Patient and public involvement	Ms	Debs	Smith	BA	UK
Researchers and Coordinators	Mr	Harry	Watson	BSc (Hons)	KCL, UK

Acknowledgments
We are grateful to all the patients and caregivers who took part in this study. We also want to acknowledge the helpful advice and input from our patient and public involvement (PPI) group members and our partners from the European Lung Foundation, ELF, and European Respiratory Society.

We thank the BETTER-B independent critical friends and advisors (some also served on oversight committees): Professor Giovanni Apolone, the late Professor Randall Curtis, Professor Daisy J.A. Janssen, Professor Dr Michael Kreuter. We thank the members of the independent data monitoring and safety committee (including Professor Magnus Ekstrom, Dr Oriana Nanni, Dr Richard Jackson, Dr Pauline Kane), trial steering committee (Dr Brian Cassel, Dr Cinzia Brunelli, Dr Sharon Love, Professor Daisy Janssen, Dr Wendy Prentice, Professor Dr Michael Kreuter, Professor Mogens Grønvold, the late Dr Sarah Booth,) and ethics advisory board (including Dr Wendy Prentice, the late Professor Randall Curtis).

We thank:

- Consortium Members - Site Principal Investigators - University of Nottingham, UK: Dr Andrew Wilcock, Calvary Healthcare Kogarah, Australia: Dr Caitlin Sheehan, UCSC, Italy: Professor Giovanni Gambassi.
- Clinical Trials Research Unit - LEEDS, UK: Mrs Claire Dimbleby, Mrs Fiona Walker, Mrs Aisha Ali.
- Health Economics – TCD, Ireland: Ms Jingjing Jiang.
- Other clinicians, including recruiting clinicians and research nurses in particular: Nottingham, UK: Dr Vincent Crosby; Hull, UK: Professor Alyn Hugh Morice, Azienda AUSL-IRCCS Reggio Emilia, Italy: Dr Alessandro Scarascia, Dr Patrizia Ruggiero, Ms Silvia Soncini, Ms Maria Taka Lovati, Ms Valeria Soffientini. Dr Francesco Menzella; UCSC, Italy: Dr Perluigi Rio; UMK, Poland: Dr Agnieszka Nowakowska-Arendt; Kings College Hospital, UK: Laura Sharp, Paramjote Kaler; Nottingham University Hospital, UK: Emma Hadfield, Cathann Manderson; Castle Hill Hospital, UK: Rachel Flockton; Mater University Hospital & UCD, Ireland: Dr Kate O'Reilly; UCD, Ireland: Dr Anna Malara.
- Co-ordinators and researchers in particular: KCL, UK: Ms Chloe Nast; KOELN, Germany: Dr Anne Pralong.
- Patient and public involvement advisors, in particular: ERS, Switzerland: Ms Valerie Vaccaro.
